# *In silico* identification of microRNAs predicted to regulate N-myristoyltransferase and Methionine Aminopeptidase 2 functions in cancer and infectious diseases

**DOI:** 10.1371/journal.pone.0194612

**Published:** 2018-03-26

**Authors:** Ranjit Chauhan, David Datzkiw, Shailly Varma Shrivastav, Anuraag Shrivastav

**Affiliations:** 1 Department of Biology, University of Winnipeg, Winnipeg, Manitoba, Canada; 2 Department of Biochemistry and Medical Genetics, University of Manitoba, Winnipeg, Manitoba, Canada; 3 Research Institute of Oncology and Hematology, CancerCare Manitoba, Winnipeg, Manitoba, Canada; Chuo University, JAPAN

## Abstract

Protein myristoylation is a key protein modification carried out by *N*-Myristoyltransferase (NMT) after Methionine aminopeptidase 2 (MetAP2) removes methionine from the amino-terminus of the target protein. Protein myristoylation by NMT augments several signaling pathways involved in a myriad of cellular processes, including developmental pathways and pathways that when dysregulated lead to cancer or immune dysfunction. The emerging evidence pointing to NMT-mediated myristoylation as a major cellular regulator underscores the importance of understanding the framework of this type of signaling event. Various studies have investigated the role that myristoylation plays in signaling dysfunction by examining differential gene or protein expression between normal and diseased states, such as cancers or following HIV-1 infection, however no study exists that addresses the role of microRNAs (miRNAs) in the regulation of myristoylation. By performing a large scale bioinformatics and functional analysis of the miRNAs that target key genes involved in myristoylation (NMT1, NMT2, MetAP2), we have narrowed down a list of promising candidates for further analysis. Our condensed panel of miRNAs identifies 35 miRNAs linked to cancer, 21 miRNAs linked to developmental and immune signaling pathways, and 14 miRNAs linked to infectious disease (primarily HIV). The miRNAs panel that was analyzed revealed several NMT-targeting mRNAs (messenger RNA) that are implicated in diseases associated with NMT signaling alteration, providing a link between the realms of miRNA and myristoylation signaling. These findings verify miRNA as an additional facet of myristoylation signaling that must be considered to gain a full perspective. This study provides the groundwork for future studies concerning NMT-transcript-binding miRNAs, and will potentially lead to the development of new diagnostic/prognostic biomarkers and therapeutic targets for several important diseases.

## Introduction

The onset of carcinogenesis is initiated by mutations to begin with in a normal cell that results in the loss of growth control (hyperplasia). Hyperplasia proceeds with the loss of senescence control, replicative immortality, apoptosis resistance and the ability to evade the immune system, which are the hallmark features of cancer and are intrinsic properties in nature [1]. Later on extrinsic factors get involved where the abnormal cancerous cell begins the process of angiogenesis (acquiring blood supply) in order to thrive, evade the surrounding tissue and colonize at distal sites (metastases) through the epithelial to mesenchyme transition. The culprit for driving such traits in the journey of a normal cell to cancer is primarily dysregulation of the signaling in the abnormal microenvironment in which these cancerous cells exists, which plays an important role in escaping the immune cells responsible for policing them. A healthy microenvironment (extracellular matrix) has the ability to suppress the cancerous growth.

**Fig 1:**
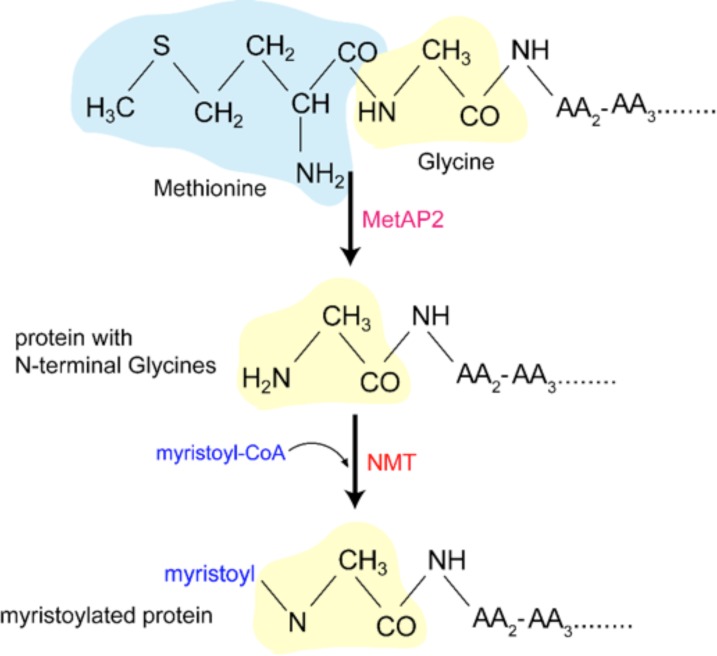
Schematic representation of protein N-myristoylation.

In order to create effective and personalized strategies for cancer treatment, it is imperative to understand the many dimensions of signaling dysregulation that characterize different types of cancer and compromised immune cells. Of the signaling molecules implicated in either immune dysfunction or cancer, N-myristoyltransferase (NMT), the enzyme responsible for the covalent attachment of a 14C myristic fatty acid to the N-terminus of target proteins, has been shown to be implicated in both the development of cancer and impaired immune cell function [2–6]. Myristoylation is preceded by the removal of the N-terminal methionine of the target protein by Methionine Aminopeptidase 2 (MetAP2) (and aids in protein trafficking directed to cellular membrane systems (Fig 1) [6]. Prokaryotes lack NMT, whereas lower eukaryotes like protozoans and fungus have a single copy of NMT, and mammals and other vertebrates, NMT has been shown to be present in two major isoforms, NMT1 and NMT2, which catalyze the same reaction, and are coded respectively by different genes [7, 8]. The biological role of NMT serves as a promising candidate to study with regards to cancer progression and immune function as its dysregulation has been shown to contribute to defective embryo and monocyte development, cell growth, T-cell signaling, and HIV infection. Little is known about the regulation of its expression, signaling, and localization [4, 5, 8–10].

The methionine residue of a nascent polypeptide is removed co-translationally by MetAP2, which is followed by addition of a myristoyl group to the exposed N-terminal glycine residue by NMT.

We are increasingly aware of the multitude of complex and intertwined signaling events occurring within a cell under various stimuli and stresses, and the many forms of regulation that govern and balance them. These events include functions prone to driving cancer, such as cell proliferation, mitosis and migration, and the respective checks in place to limit growth. At the heart of cell signaling and regulation is the myriad of genes that make up the genome, and their respective protein end products that serve to exert specific cellular functions. However, the human genome, once estimated to contain over 100, 000 protein coding genes, has been re-evaluated over the years to be comprised of merely ~20, 000 distinct genes, which in turn code for an estimated 293, 000 non-redundant peptides [11, 12]. This revelation has led researchers to seek and shed light on the various forms of genomic regulation that influence gene expression, fine-tune spatio-temporal aspects of cell signaling, and account for the massive repertoire of distinct peptides. To date, several regulatory systems have been identified and well established, including transcriptional regulation through various pleiotropic transcription-factor family proteins, and epigenetic methylation of DNA, as well as post-translational mechanisms of gene regulation, such as increased proteome diversity through mRNA splicing mediated by the spliceosome, and gene downregulation *via* mRNA silencing by various RNA products [13–16]. Of the various agents responsible for RNA silencing, there has been emerging evidence pointing to the importance of microRNAs (miRNAs) in several facets of cellular functional regulation, including those involved in cancer progression and immune function [17–19].

MicroRNAs (miRNAs) are a family of endogenously expressed small non-coding single stranded RNAs that are generally 21–23 nucleotides in length [20]. The maturation of miRNA in humans is facilitated by two consecutive cleavages mediated by RNAIII enzymes DROSHA and DICER. miRNAs are the final product of larger pri-mRNAs, the latter transcribed by either RNA polymerase II or III. DROSHA complexes along with its partner DiGeorge syndrome critical region 8 (DGCR8) in a stoichiometry of 1:2 respectively form the microprocessor complex. The microprocessor complex reduces the pri-mRNA to an ~85 nucleotide long pre-miRNA containing a hairpin loop [21, 22]. Pre-miRNA is transported into the cytoplasm, recruited to the spliceosome, and subsequently cleaved by the DICER enzyme to yield a mature miRNA duplex [23]. One of the strands of the cytoplasmic miRNA, known as guide is used to target miRNA onto a protein named Argonaute to form the RNA-induced silencing complex (RISC). RISC is a multi-protein complex that is guided by the sequence of miRNA transcript to a target (through complementary base pairs) and the protein Argonaute cleaves mRNA (messenger RNA) [24]. Thus, miRNA is capable of causing degradation of the target mRNA if perfect nucleotide complementation is achieved, otherwise translational repression of the target mRNA occurs in the case of imperfect complementarity [25]. miRNA have been shown to have a certain degree of genomic organization, adding an additional layer of complexity to miRNA systems that can be manipulated to drive evolution and specialization [26]. Some miRNAs have been shown to form polycistronic clusters that in some cases co-express several miRNAs that target different mRNAs responsible for proteins within the same protein complex [27, 28]. These findings demonstrate the ability of miRNA to influence protein-protein interactions.

The repertoire of miRNA that can be expressed by a cell constitutes an essential layer of post-transcriptional gene control for many cellular processes. miRNAs are relatively young on the evolutionary timescale, being expressed only in animals, plants, and some viruses [29–31]. Around 30% of the *Homo sapiens* protein-coding genes are regulated by miRNAs, which control the genes at the post-transcriptional level [32]. There are two modes by which miRNAs regulates the expression of genes; first, miRNA-mediated transcript degradation and second, inhibition of protein translation [25, 33]. For target degradation model, miRNA binds predominantly to the target sequence found within the 3’ untranslated region (UTR) of the target mRNA with perfect complementarity, leading the mRNA to be cleaved [34, 35]. Similarly to inhibit the translation of target genes, miRNA binds with imperfect complementarity with the target. However, recent studies suggest that even with the imperfect complementarity between miRNA and target mRNA sequences, miRNAs are capable of carrying out target recognition and subsequent translational inhibition and/or transcriptional decay [36]. In addition to its functions in post-transcriptional gene regulation, miRNAs are also known for regulating protein complexes and acting as a key-determining molecule in protein-protein interaction [27, 28].

The role of miRNA has been suggested to be more of a fine-tuning mechanism of gene regulation, rather than as a master regulator; however, increasing evidence has shown that miRNAs are heavily dysregulated in many diseases, including cancer [37–40]. There are several studies linking microRNA as a driving factor in the progression of some cancers, such as the promotion of colorectal cancer proliferation and invasion by miR-320b [41]. In some cases miRNA can act as a tumor suppressor, such as miR-29c, which is correlated with breast cancer survival and downregulates B7-H3 protein which is associated with metastasis and poor prognosis in breast cancer patients [42]. Beyond the potential of miRNAs to play a positive or negative role in disease, they may serve to act as novel biomarkers [43]. Increasing evidence is revealing that specific circulating miRNAs may be used as non-invasive biomarkers for neoplastic diseases, such as breast cancer (miR-29c, 199a, 424) [44], colorectal cancer (miR-24, 320a, 423-5p) [45], and liver cancer (miR-200 family) as well as its regression (miR-199a-3p) [46, 47]. In cancer biology, miRNAs may be playing a critical role by modulating key signaling pathways, as they have been shown to affect the sensitivity of a cell to signal transduction by signaling molecules such as epidermal growth factor, Notch, TGF- β, and WNT [48, 49].

We suspect that the role of critical signaling modulation by miRNAs can be extended to their interactions with NMT translation and influence over protein-protein interaction related to NMT signaling. Dysregulation of NMT1 activity is implicated in cancer and stem cell differentiation [8, 50]. The plasticity of transition from normal to cancerous cells as well as stem cell differentiation and proliferation to T lymphocytes depend on the miRNA target gene regulation [4, 51]. In this study we predicted the miRNAs that target NMT1/NMT2/MetAP2 transcripts, and the possibility of these interactions to inhibit NMT1/2 expression, and their function in relation to cancer, stem cell, T-cell/B-cell signaling and infectious diseases, using the bioinformatics techniques TargetScan 7 and DIANA.

## Materials and methods

### Sequence selection for computational analysis

For *NMT1* and *NMT2* reference sequences were retrieved from the GenBank. *NMT1* and *NMT2* reference numbers were NM_021079.4 and NM_004808, respectively. For *MetAP2*, the reference sequence used was NM_001317182.1.

### Prediction of miRNA targets

The putative miRNA targets were predicted using two annotation programs as described previously [52]. Prediction of miRNAs was done in the subtractive library. The sequences were submitted for *in silico* annotation of ncRNAs. During the annotation process, we searched for the RNA structures by using Infernal (INFERence of RNA ALignment) software as described in other study [53]. The BLAST program was used to search similar miRNA sequences in the National Center for Biotechnology Information (NCBI) database as described elsewhere [54]. Finally, miRNAs with the best *p*-value (≤0.05) were selected for further analysis and their details are presented in tabulated form (S1 Table).

### Identification and functional annotation of miRNAs regulating NMT and MetAP2 genes using TargetScan and DIANA Tools mirPath

Sequences for human *NMT1/2* and *MetAP2* genes (18 and 11 transcripts respectively) were downloaded from the ENSEMBL genome browser (*NMT*: ENSG00000136448, *MetAP2*: ENSG00000111142). Sequences of human mature miRNAs (2,588) were downloaded from miRBase version 21 (http://mirdb.org) [55]. Normalized mRNA and miRNA expression values were downloaded from Gene Expression Omnibus (GEO) repository (accession ID: GSE62030). miRNAs targeting *NMT1/2* and *MetAP2* genes were predicted by TargetScan7 (v7.0; targetscan.org) [56]. To validate the predicted miRNA: target interactions, Pearson correlation coefficient (PCC) was calculated using the normalized expression values of miRNA and target genes. All the miRNA:target interactions having significantly high inverse/negative PCC (*r < -0*.*5*, *p ≤ 0*.*05*) were considered as true miRNA:target interactions. The *p*-value of PCC was calculated by Student’s t-test using “R” software. Biological pathways influenced by miRNAs targeting *NMT* and *METAP2* genes were identified using DIANA Tools mirPath version 3 (v3.0) (http://www.microrna.gr/miRPathv3) [57].

### Functional enrichment analysis of miRNA targets

To determine or predict the function(s) of miRNAs which targets these genes, pathway enrichment analysis was performed using DIANA Tools mirPath (v3.0) as described previously [57]. This tool provides information on experimentally-supported miRNA functional annotation using Gene Ontology (GO) or GOSlim terms [58], combined with statistically-enriched pathways, mainly Kyoto Encyclopedia of Genes and Genomes (KEGG) molecular pathways, and based on target genes which query miRNAs targets [57].

### Literature search for miRNAs associated with cancer and signaling pathways

The chosen set of miRNAs from bioinformatics analyses were thoroughly researched in the literature for their association, functions and expression changes in cancer and signaling pathways. PubMed, PubMed Central and Google Scholar search engines were utilized to perform the literature survey. MiRNAs that showed at least one publication record were considered for further analysis in the study.

### MiRNA clustering analysis

MiRNA expression values were extracted from “GSE62037” GEO dataset [59]. The miRNAs that were predicted to target *NMT1/2* transcripts were filtered out and their expression values were specifically extracted from the above dataset. In order to recognize the normalized expression patterns of these miRNAs, an unsupervised hierarchical cluster analysis were carried out using Cluster v3.0 as described elsewhere [60]. TreeView software was used to generate and visualize the heatmaps. Green color shows positive PCC values (0.5 ≤ *r* ≤ 1) and red color shows inverse/negative PCC values (-0.5 ≤ *r* ≤ -1). Thereafter, it was determined whether the miRNA data fit into known post-transcriptional ‘RNA regulon (operon) model’ which describes how RNA molecules are organized at a higher-organization level and how their functional dynamics are connected to post-transcriptional regulatory events such as stability and translation [61, 62].

## Results

### Identification of miRNAs targeting NMT and METAP2 genes

Mature miRNAs targeting 18 transcripts and 11 transcripts of *NMT1/2* and *MetAP2* respectively, were identified using TargetScan7 [63]. Using the stringent cutoff, a total of 13,798 miRNA-target interactions were predicted by TargetScan for the *NMT1/2* genes. In contrast, 7,708 interactions were predicted for the *MetAP2* gene.

In order to filter out false positive miRNA: target interactions, Pearson correlation coefficient (PCC) was calculated between targeting miRNA and target gene expression. In general, miRNAs down-regulates the expression of a target gene. Based on previous reports, the threshold PCC for determining the true positive miRNA: target interaction was set at 0.5 [64, 65]. Thus, a true interaction will be indicated by significantly high inverse PCC (*r ≤ -0*.*5*, *p ≤ -1*). Out of 13,798 putative miRNAs: target interactions predicted for the *NMT1/2* genes, only 221 miRNAs: target interactions showed *r ≤ -0*.*5* (S1 Table). The top five miRNAs targeting the *NMT1/2* genes with the highest inverse PCC were *miR-421*, *miR-4317*, *miR-606*, *miR-140-5p* and *miR-941*. Interestingly, these miRNAs were also found to be regulating multiple *NMT1/2* transcripts (S1 Table). Similarly, for the *METAP2* gene, out of 7,708 interactions predicted, 165 miRNAs: target interactions showed PCC values above our cutoff threshold (*r ≤ -0*.*5*) (S2 Table). Based on the PCC values the top five miRNAs targeting the *MetAP2* gene turned out to be *miR-330-3p*, *miR-421*, *miR-409-3p*, *miR-139-3p*, and *miR-1246* (S2 Table).

**Fig 2:**
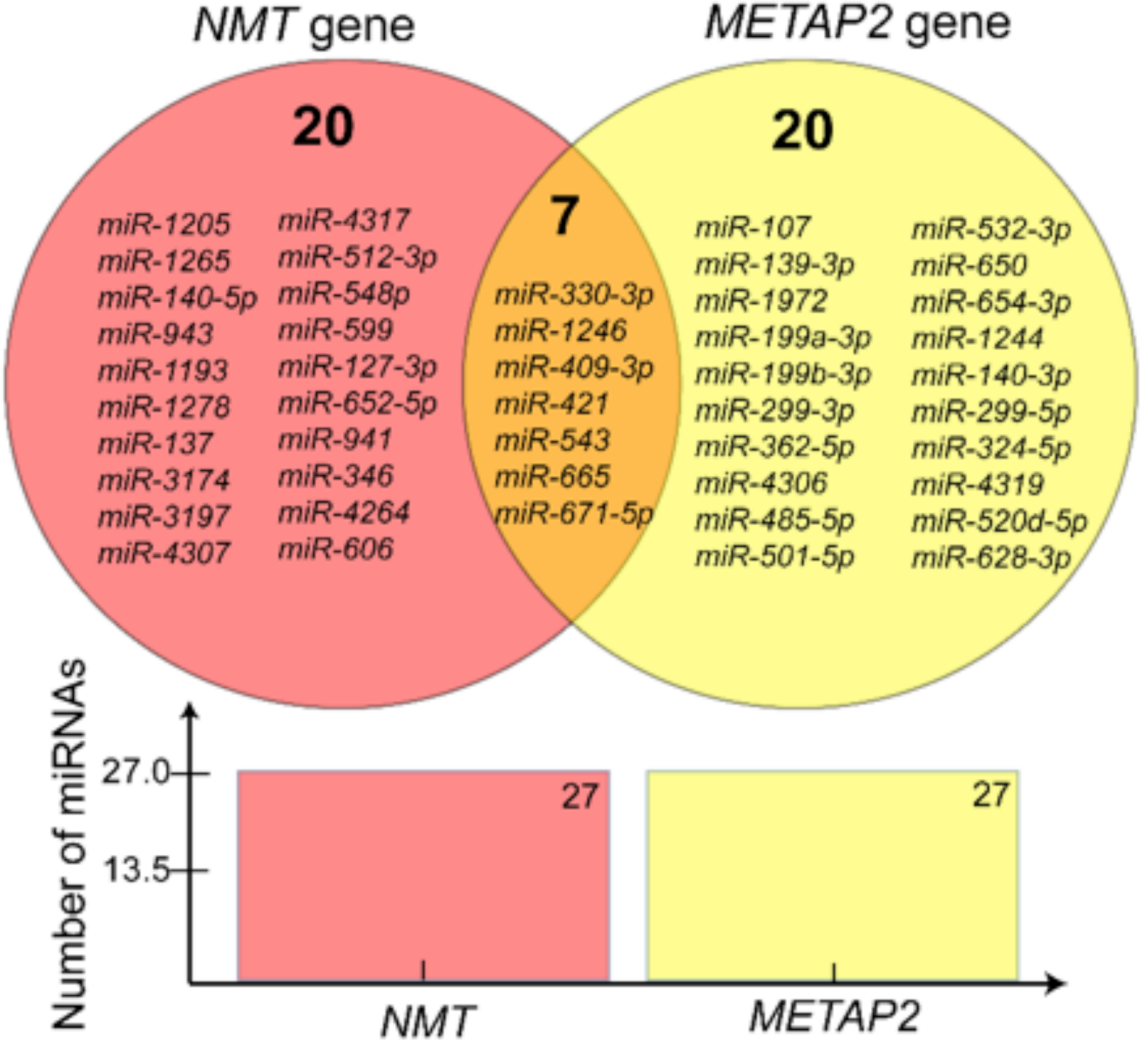
The distribution of microRNAs (miRNAs) that target N-myristoyltransferase (NMT1/2) and methionine aminopeptidase 2 (MetAP2) genes. The miRNAs that target either NMT or MetAP2, or both genes are illustrated in the Venn diagram. Seven miRNAs target both *NMT1/2* and *MetAP2* genes.

Next, to determine whether same miRNA targets *NMT1/2* and *MetAP2* genes, we compared the targeting miRNAs lists (S1 and S2 Tables). The results revealed that 7 miRNAs are common which targets *NMT1/2 and MetAP2* genes while 20 miRNAs were found to be exclusively targeting either *NMT1/2* or *MetAP2* genes (Table 1; Fig 2). From the list of commonly targeting miRNAs, *miR-421* has the highest inverse co-expression with the target genes and appeared in the top five miRNAs. Making this miRNA a top candidate for further evaluation.

**Table 1:** MicroRNAs (miRNAs) that are specific and common to N-myristoyltransferase (*NMT1/2*) and methionine aminopeptidase 2 *(MetAP2)* genes.

	miRNAs targeting *NMT1/2*	miRNAs targeting *MetAP2*	Common miRNAs
1	*miR-1205*	*miR-107*	*miR-330-3p*
2	*miR-1265*	*miR-139-3p*	*miR-1246*
3	*miR-140-5p*	*miR-1972*	*miR-409-3p*
4	*miR-943*	*miR-199a-3p*	*miR-421*
5	*miR-1193*	*miR-199b-3p*	*miR-543*
6	*miR-1278*	*miR-299-3p*	*miR-665*
7	*miR-137*	*miR-362-5p*	*miR-671-5p*
8	*miR-3174*	*miR-4306*	
9	*miR-3197*	*miR-485-5p*	
10	*miR-4307*	*miR-501-5p*	
11	*miR-4317*	*miR-532-3p*	
12	*miR-512-3p*	*miR-650*	
13	*miR-548p*	*miR-654-3p*	
14	*miR-599*	*miR-1244*	
15	*miR-127-3p*	*miR-140-3p*	
16	*miR-652-5p*	*miR-299-5p*	
17	*miR-941*	*miR-324-5p*	
18	*miR-346*	*miR-4319*	
19	*miR-4264*	*miR-520d-5p*	
20	*miR-606*	*miR-628-3p*	

### Functional enrichment analysis of miRNA targets

To identify the biological pathways under regulation of the miRNAs identified in the previous section, functional enrichment was done using DIANA Tools mirPath version 3 [57]. The statistically significant KEGG pathways enriched from the analyses are summarized in Table 2. The analysis revealed 88 pathways that belong under the KEGG pathways. It was found that the most significantly enriched pathway regulated by miRNAs that target both *NMT1/2* genes and the *MetAP2* gene was proteoglycans in cancer. Interestingly, one pathway regulated by the selected miRNAs is the ErbB signaling pathway. This pathway plays an important role in regulating cancer [66]. Furthermore, Wnt, mTOR, and VEGF signaling pathways were found to be regulated by miRNAs that target *NMT* gene. Additionally, we also predicted KEGG pathways that are associated with the miRNAs that exclusively targets *NMT1/2* or *MetAP2* genes. From these pathways, the most significant ones were filtered for further exploration in terms of their relevance in cancer.

**Table 2:** KEGG pathways enrichment annotation of the microRNAs (miRNAs) that target N-myristoyltransferase (*NMT1/2*) and methionine aminopeptidase 2 *(MetAP2)* genes.

	KEGG pathway	*p*-value	#genes	#miRNAs
1	ErbB signalling pathway	3.60E-29	48	17
2	Prostate cancer	8.54E-27	46	17
3	Colorectal cancer	8.44E-23	36	17
4	Wnt signalling pathway	1.88E-21	71	17
5	mTOR signalling pathway	6.21E-21	36	14
6	Long-term potentiation	1.03E-20	36	12
7	VEGF signalling pathway	1.38E-19	34	16
8	Pancreatic cancer	5.86E-19	36	17
9	Focal adhesion	1.04E-18	83	16
10	Endometrial cancer	1.20E-17	29	16
11	Non-small cell lung cancer	2.04E-16	28	14
12	Neurotrophin signalling pathway	4.14E-16	54	17
13	MAPK signalling pathway	6.31E-16	100	17
14	Insulin signalling pathway	1.79E-15	58	17
15	B cell receptor signalling pathway	9.73E-15	36	17
16	TGF-beta signalling pathway	2.47E-14	39	15
17	Acute myeloid leukemia	7.30E-14	29	14
18	Axon guidance	2.30E-13	57	16
19	Dopaminergic synapse	2.60E-12	54	16
20	PI3K-Akt signalling pathway	3.19E-12	118	17
21	Glioma	3.82E-12	35	14
22	Long-term depression	3.85E-12	34	12
23	Pathways in cancer	4.64E-12	125	17
24	Chronic myeloid leukemia	7.05E-11	34	16
25	Melanoma	9.58E-11	32	14
26	Gap junction	1.33E-10	38	15
27	Aldosterone-regulated sodium reabsorption	6.90E-10	19	11
28	Renal cell carcinoma	3.82E-09	34	16
29	Regulation of actin cytoskeleton	4.15E-09	82	16
30	HIF-1 signalling pathway	4.40E-09	44	15
31	mRNA surveillance pathway	5.38E-09	38	16
32	T cell receptor signalling pathway	6.87E-09	43	18
33	Circadian rhythm	3.95E-08	15	9
34	GnRH signalling pathway	1.24E-07	36	14
35	Prion diseases	3.83E-07	11	7
36	RNA degradation	3.83E-07	30	11
37	Retrograde endocannabinoid signalling	3.99E-07	46	16
38	Protein processing in endoplasmic reticulum	8.13E-07	64	15
39	Thyroid cancer	1.10E-06	14	11
40	Cholinergic synapse	2.16E-06	46	17
41	Fc gamma R-mediated phagocytosis	3.37E-06	36	16
42	Progesterone-mediated oocyte maturation	4.67E-06	33	17
43	Shigellosis	5.15E-06	27	12
44	Hedgehog signalling pathway	6.01E-06	21	15
45	Small cell lung cancer	6.63E-06	32	14
46	Hepatitis B	1.10E-05	55	17
47	Melanogenesis	1.15E-05	38	16
48	Fc epsilon RI signalling pathway	1.47E-05	28	16
49	Adherens junction	1.68E-05	32	15
50	Glutamatergic synapse	2.85E-05	45	16
51	Ubiquitin mediated proteolysis	3.98E-05	48	14
52	Bacterial invasion of epithelial cells	6.28E-05	29	12
53	Phosphatidylinositol signalling system	7.57E-05	32	14
54	Viral myocarditis	9.57E-05	26	14
55	Calcium signalling pathway	1.46E-04	60	17
56	Type II diabetes mellitus	1.89E-04	19	13
57	HTLV-I infection	1.96E-04	86	18
58	Tight junction	2.10E-04	48	17
59	p53 signalling pathway	2.17E-04	27	14
60	Hepatitis C	2.50E-04	45	17
61	Chemokine signalling pathway	3.14E-04	61	17
62	Transcriptional misregulation in cancer	4.00E-04	61	17
63	Hypertrophic cardiomyopathy (HCM)	4.82E-04	30	14
64	Osteoclast differentiation	7.03E-04	44	16
65	Vascular smooth muscle contraction	8.81E-04	42	15
66	Endocrine and other factor-regulated calcium reabsorption	2.18E-03	20	10
67	Dilated cardiomyopathy	2.56E-03	31	16
68	Protein digestion and absorption	2.65E-03	30	13
69	Epithelial cell signalling in Helicobacter pylori infection	2.90E-03	24	12
70	Basal cell carcinoma	2.90E-03	20	16
71	RNA transport	4.84E-03	50	17
72	Nicotine addiction	4.87E-03	18	11
73	Amoebiasis	7.13E-03	36	14
74	Chagas disease (American trypanosomiasis)	7.19E-03	36	17
75	Jak-STAT signalling pathway	7.39E-03	48	16
76	Arrhythmogenic right ventricular cardiomyopathy (ARVC)	9.00E-03	29	14
77	Serotonergic synapse	9.00E-03	37	15
78	Apoptosis	1.24E-02	32	15
79	Adipocytokine signalling pathway	1.31E-02	23	11
80	Inositol phosphate metabolism	1.92E-02	21	14
81	Gastric acid secretion	2.73E-02	25	12
82	Oocyte meiosis	3.01E-02	42	15
83	ABC transporters	3.47E-02	15	10
85	NOD-like receptor signalling pathway	3.82E-02	19	12
86	Salivary secretion	4.10E-02	28	13
87	Fanconi anemia pathway	4.91E-02	18	12
88	Endocytosis	4.91E-02	62	16

**Fig 3:**
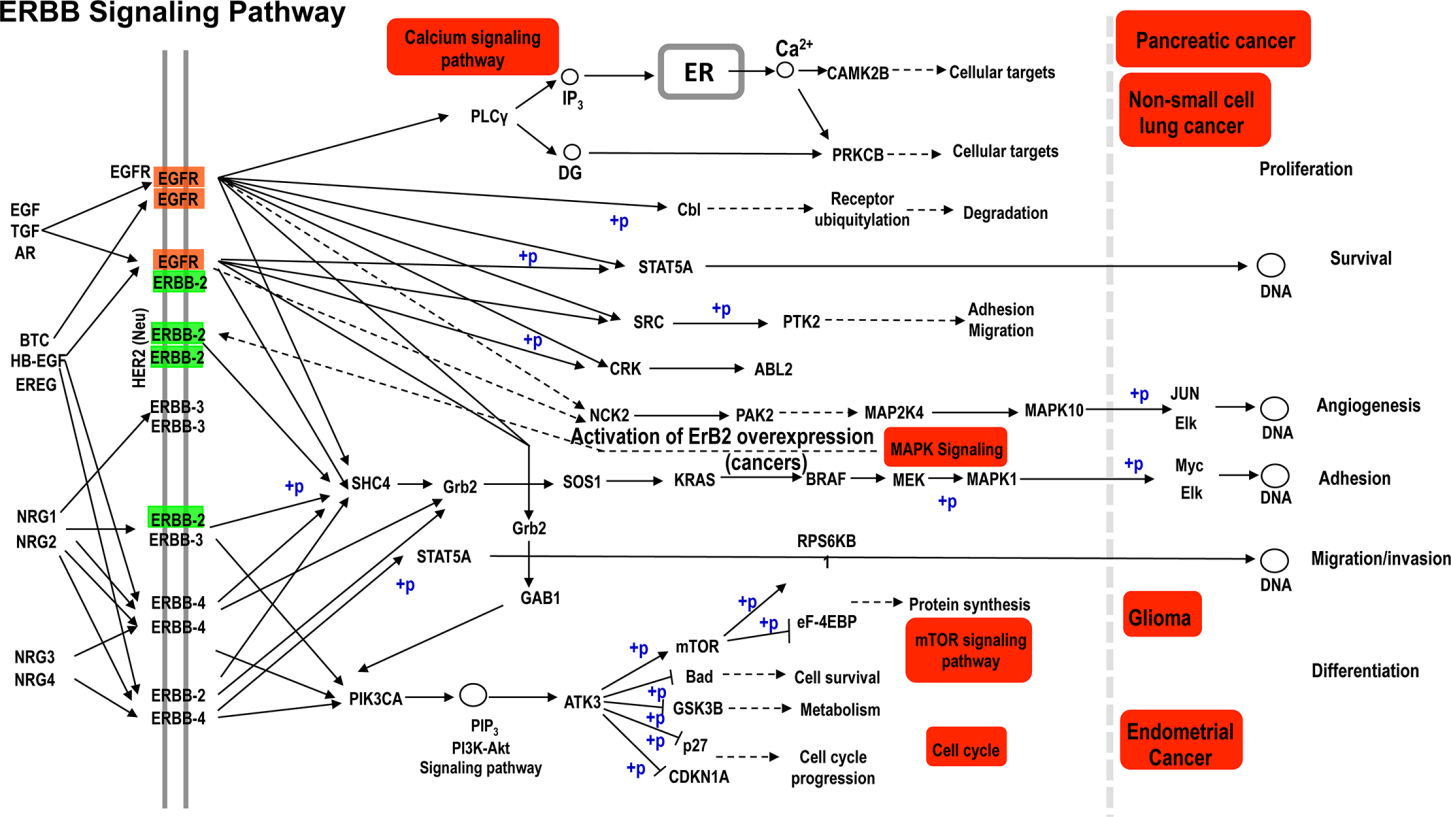
ErbB signaling pathway that is enriched with target genes of microRNAs (miRNAs) which negatively regulate N-myristoyltransferase *(NMT1/2)* and methionine aminopeptidase 2 *(MetAP2)* genes. The figure illustrates ErbB signaling pathway that contain genes that are targeted by miRNAs which regulate *NMT* gene. (EGF, epidermal growth factor; TGF, transforming growth factor; BTC, betacellulin; HB-EGF, heparin-binding epidermal growth factor (EGF)-like growth factor; EREG, epiregulin; NRG1, neuregulin-1; NRG2, neuregulin-2; NRG3, neuregulin-3; NRG4, neuregulin-4; PLCγ, phospholipase C type gamma; CAMK2B, calcium/calmodulin dependent protein kinase; PRKCB, Protein kinase C-beta; STAT5, Signal transducer and activator of transcription 5; src, Rous sarcoma virus gene; CRK, C T10 regulator of a tyrosine kinase; NCL, NCK Adaptor Protein 2; PTK2, PTK2 protein tyrosine kinase 2; ABL2, V-Abl Abelson Murine Leukemia Viral Oncogene Homolog 2; PAK2, P21 (RAC1) Activated Kinase 2; MAP2K4, Mitogen-Activated Protein Kinase Kinase 4; MAPK10, Mitogen-Activated Protein Kinase 10; SOS1, SOS Ras/Rac Guanine Nucleotide Exchange Factor 1; Grb2, Growth Factor Receptor Bound Protein 2; SHC4, Src Homology 2 Domain-Containing-Transforming Protein C4; PIK3C4, Phosphatidylinositol-4,5-Bisphosphate 3-Kinase Catalytic Subunit; AKT3, KT Serine/Threonine Kinase 3; mTOR, Mechanistic Target Of Rapamycin Kinase; BCL2, BCL2 Associated Agonist Of Cell Death; GSK3B, Glycogen Synthase Kinase 3 Beta; CDKN1A, Cyclin Dependent Kinase Inhibitor 1A; EIF4EBP1, Eukaryotic Translation Initiation Factor 4E Binding Protein 1; BRAF, B-Raf Proto-Oncogene, Serine/Threonine Kinase; RPS6KB1, Ribosomal Protein S6 Kinase B1; KRAS, KRAS Proto-Oncogene, GTPase; JUN, Jun Proto-Oncogene, AP-1 Transcription Factor Subunit; ELK, ETS Transcription Factor; Myc, MYC Proto-Oncogene, BHLH Transcription Factor; ER, endoplasmic reticulum. DNA, deoxyribonucleic acid).

The DIANA Tools mirPath analysis predicted that most significantly enriched KEGG pathway regulated by miRNAs targeting both *NMT* and *MetAP2* genes was ‘ErbB signaling pathway’ (*p = 3*.*60E-29*) which involved 48 genes and 17 miRNAs (Table 2; Fig 3). ErbB receptor molecules regulate cell proliferation, differentiation, cell motility, and cell survival. Therefore, ErbB receptor mutations or overexpression have been associated with cancer cell migration, development, invasion and progression of cancers such as non-small cell lung cancer [67], breast cancer [68], ovarian cancer and bladder cancer [69]. This is mainly due to the role of this pathway in phosphorylation of many important kinases involved in cancer pathology.

**Fig 4:**
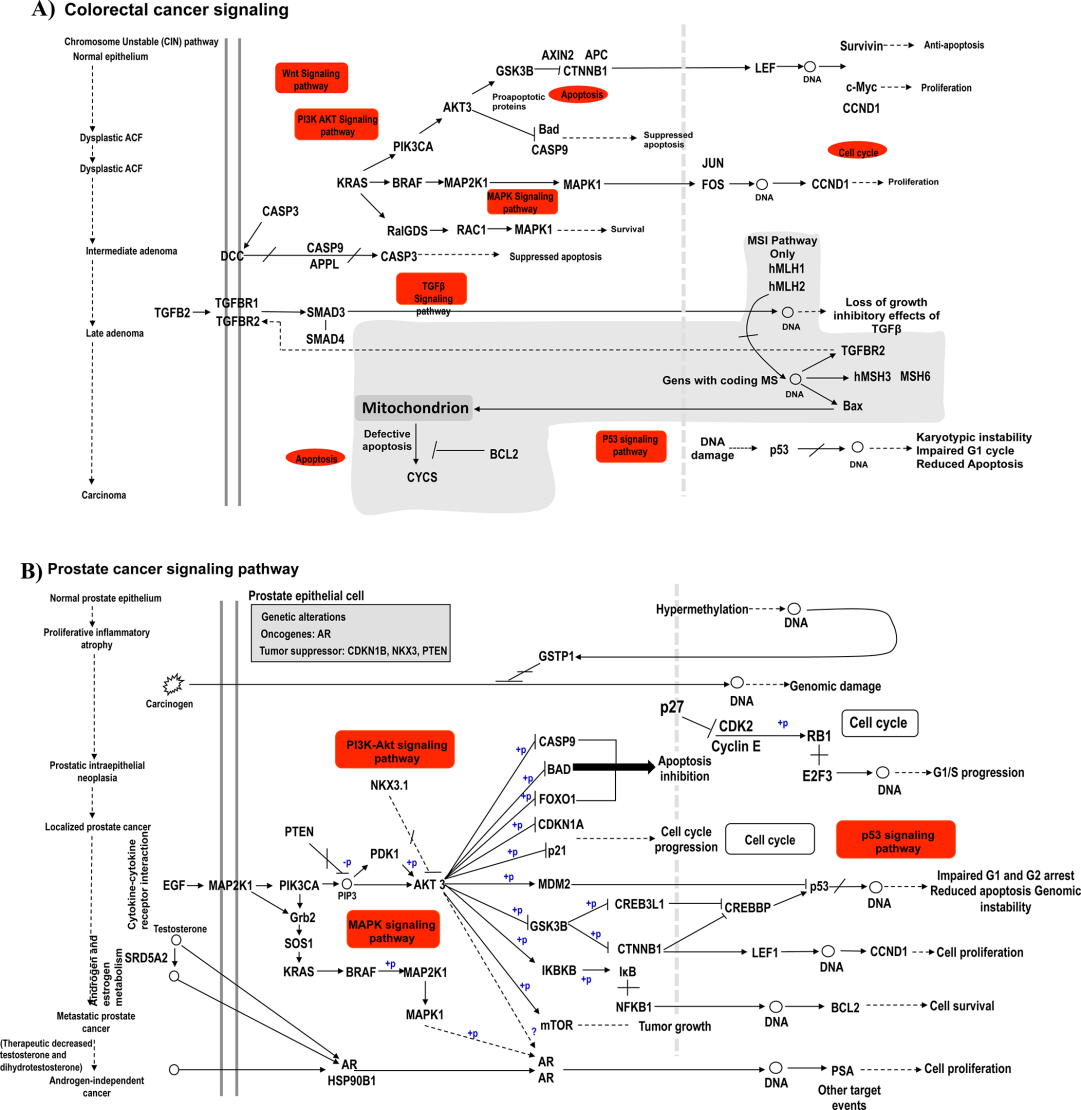
Colorectal cancer and prostate cancer pathways that are enriched with target genes of microRNAs (miRNAs) which negatively regulate N-myristoyltransferase *(NMT1/2)* and methionine aminopeptidase 2 *(MetAP2)* genes. The figure illustrates **A)** colorectal cancer pathway and **B)** prostate cancer pathway that contain genes that are targeted by miRNAs which regulate *NMT* gene. (Rac1, Rac family small GTPase 1; Wnt, Wingless-related integration site; FOS, Fos Proto-Oncogene, AP-1 Transcription Factor Subunit; APC, Adenomatosis Polyposis Coli Tumor Suppressor; AXIN2, Axis Inhibition Protein 2; CTNNB1, Catenin Beta 1; CASP3, Caspase 3; APPL, Adaptor Protein, Phosphotyrosine Interacting With PH Domain And Leucine Zipper 1; CCND1, Cyclin D1; RALGDS, Ral Guanine nucleotide dissociation stimulator; MSH6, MutS homolog 6; BCL2, B-Cell CLL/Lymphoma 2; TGFBR2, Transforming Growth Factor Beta Receptor 2; SMAD, SMAD Family Member 3 (mothers against decapentaplegic); Myc, MYC Proto-Oncogene; BAX, BCL2 Associated X, Apoptosis Regulator; CYCS, Cytochrome C, DCC, Deleted In Colorectal Carcinoma; LEF, lymphoid enhancer binding factor 1; P53, Phosphoprotein-53. AR, androgen receptor; FOXO1, Forkhead Box O1; PTEN, Phosphatase And Tensin Homolog; GSTP1, Glutathione S-Transferase Pi 1; CDK2, Cyclin Dependent Kinase 2; Rb1, Retinoblastoma 1; E2F3, E2F Transcription Factor 3; EGF, Epidermal Growth Factor; SRD5A2, Steroid 5 Alpha-Reductase 2; PSA, Kallikrein 3; MDM2, RING-Type E3 Ubiquitin Transferase Mdm2; PDK1, Pyruvate Dehydrogenase Kinase 1; CDKN1A, Cyclin Dependent Kinase Inhibitor 1A; CREBBP, CREB Binding Protein; CREB3L1, AMP Responsive Element Binding Protein 3 Like 1).

When analyzing pathways associated with colorectal (Fig 4A) and prostate cancers (Fig 4B), we observed that miRNAs targeting both *NMT1/2* and *MetAP2* genes also regulate the expression of key genes involved in these pathways, including *AKT1*, *GSK3B*, *BRAF*, *MAPK* and many others (Fig 4).

**Fig 5:**
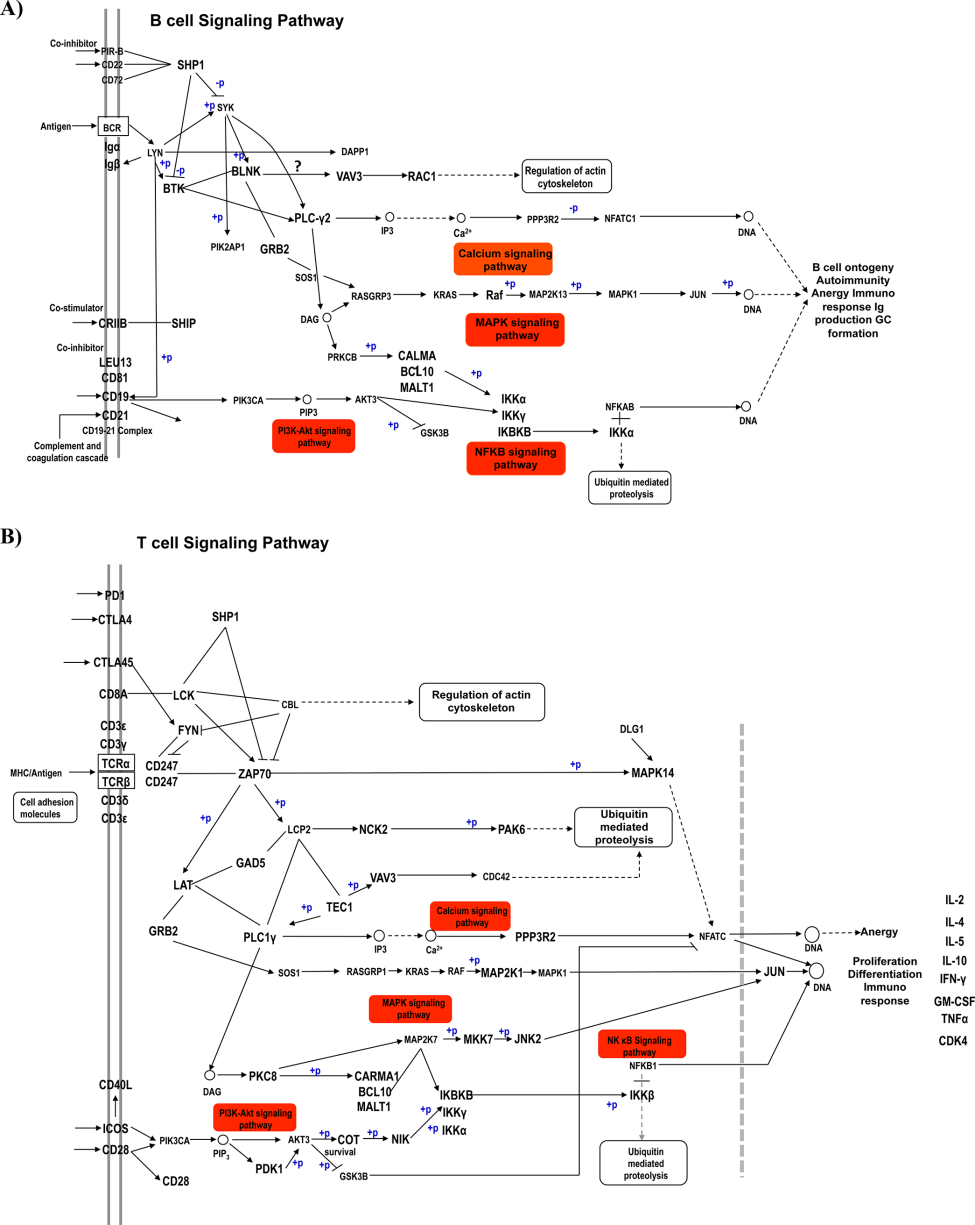
T-cell and B-cell receptor pathways that are enriched with target genes of microRNAs (miRNAs) which negatively regulate N-myristoyltransferase *(NMT1/2)* and methionine aminopeptidase 2 *(MetAP2)* genes. The figure illustrates two cellular pathways **A)** T cell receptor signaling and **B)** B cell receptor signaling that contains genes that are targeted by miRNAs, which regulate *NMT1/2* gene. (PD1, programmed cell death-1; ZAP70, Zeta Chain Of T-Cell Receptor Associated Protein Kinase 70; LAT, Linker for Activation Of T-Cells; ICOS, Inducible T-Cell Co-stimulator; DLG1, Discs Large MAGUK Scaffold Protein 1; NCK2, NCK Adaptor Protein 2; LCP2, Lymphocyte Cytosolic Protein 2; GM-CSF, Granulocyte-macrophage colony-stimulating factor; IFN-γ, Interferon gamma; TNFα, Tumor Necrosis Factor alpha; IL, Interleukin; NFKB1, Nuclear Factor Kappa B Subunit 1; CDK4, Cyclin Dependent Kinase 4; PPP3R2, Protein Phosphatase 3 Regulatory Subunit B, Beta; CTLA4, Cytotoxic T-lymphocyte Associated Protein; PKC8, protein kinase C-8; FYN, FYN Proto-Oncogene, Src Family Tyrosine Kinase; Raf-1, Raf-1 Proto-Oncogene, Serine/Threonine Kinase. CD, cluster of differentiation; BCR, break-point cluster region; BTK, Bruton Tyrosine Kinase; DAPP1, Dual Adaptor Of Phosphotyrosine And 3-Phosphoinositides 1; VAV3, Vav Guanine Nucleotide Exchange Factor 3; SHIP, SH2 Domain-Containing Inositol 5-Phosphatase; Rac1, Rac Family Small GTPase 1; SYK, Spleen Associated Tyrosine Kinase; NFATC1, Nuclear Factor of activated T-cells 1; PPP3R2, Protein Phosphatase 3 Regulatory Subunit B, Beta; RASGRP3, RAS Guanyl Releasing Protein 3; MALT1, Mucosa Associated Lymphoid Tissue Lymphoma Translocation; Lyn, LYN Proto-oncogene src family tyrosine kinase).

Apart from cancer pathways, miRNAs were also shown to regulate genes in immune cells. Therefore, we further analyzed KEGG pathways that are associated with immune responses. For this, we performed pathway enrichment analysis using miRNAs that target only *NMT* transcripts. Interestingly, T cell receptor signaling pathway (*p = 6*.*87E-09*) involving 43 genes and 18 miRNAs (Table 2; Fig 5A); and B cell receptor signaling pathway (*p = 9*.*73E-15*) involving 36 genes and 17 miRNAs were predicted by the analysis (Table 2; Fig 5B). A total of 11 and 8 miRNAs that target *NMT1* and *NMT2*, respectively were identified to be associated with T cell and B cell receptor pathways (Table 3). Among these miRNAs, *miR-654* displayed an interesting relationship with *NMT1/2* and *MetAP*2 genes where *miR-654-5p* targeted *NMT1/2* while *miR-654-3p* targeted *MetAP2*. Similarly, *miRNA-199b-5p* was found to be targeting *NMT1/2* while *miRNA-199b-3p* targeted *MetAP2*. This 5p and 3p pattern was not limited to these two miRNAs. Same pattern was observed with *miR-628*, and *miRNA-139*. These observations suggest that the miRNA species deriving from the 5' arm (5p) and 3' arm (3p) of the same pre-miRNA can regulate both *NMT1/2* and *MetAP2* genes depending upon which mature miRNA is loaded on to Argonaute protein. Additionally, *miR-1246* was also common between *NMT1/2* and *MetAP2* genes.

**Table 3:** MicroRNAs (miRNAs) that target T cell and B cell signaling pathways and stem cell signaling.

	*NMT1* targeting miRNAs	Specific	Up/Down Regulated	References
1	*miRNA-652-5p*	Monocytes	Downregulated	[70]
2	*miRNA-27a-3p*	Dendritic cells	Upregulated	[71]
3	*miRNA-654-5p*	Mesenchymal Stem Cells	Upregulated	[72]
4	*miRNA-379-5p*	Granulocytes	Downregulated	[73]
5	*miRNA-512-3p*	T Cells	Regulatory role	[74]
6	*miRNA-181c-5p*	T Cells	Upregulated	[75]
7	*miRNA-3174*	Mesenchymal Stem Cells	Regulatory role	[76]
8	*miRNA-200b-3p*	Monocytes	Regulatory role	[77]
9	*miRNA-326*	T_H_-17 Differentiation	Regulatory role	[78]
10	*miRNA-29b-1-5p*	T cells	Regulatory role	[79]
11	*miRNA-148b-5p*	DC/T Regulatory Cells	Regulatory role	[80]
	***NMT2* targeting miRNA**			
1	*miRNA-199b-5p*	Stem Cells	Regulatory	[81]
2	*miRNA-628-5p*	GMCSF	Regulatory	[82]
3	*miRNA-1246*	T Regulatory Cells	Upregulated	[83]
4	*miRNA-599*	T Cells	Regulatory	[84]
5	*miRNA363-5p*	Stem Cells	Upregulated	[85]
6	*miRNA-26a-5p*	B cells	Downregulated	[86]
7	*miRNA-139-5p*	Stem cells	Regulatory	[87]
8	*miRNA-3197*	Stem Cell	Downregulated	[88]

Further analysis of the KEGG pathways enriched with miRNAs that targets only *NMT1/2* gene transcripts revealed signaling pathways that regulate the pluripotency of stem cells, Hippo signaling pathway, estrogen signaling pathway and glioma. On the other hand, a similar analysis for miRNAs that target *MetAP2* gene revealed regulation of fatty acid biosynthesis, signaling pathways regulating pluripotency of stem cells, ErbB signaling pathway and prion diseases as the top KEGG pathways involved. Interestingly, all these KEGG pathways are related to the genes/proteins that regulate cellular growth, survival, migration, proliferation and development, which are basically essential cellular processes involved in cancer progression [62]. Taken together, the predicted data strongly support the association of miRNAs targeting *NMT1/2* and *MetAP2* genes to signaling pathways implicated in cancer and immune response.

### Further analysis of signaling networks

#### Cancer signaling

We mined data to identify the roles of 221 miRNAs that regulate NMT in cancer. We found that 35 (15.8%) miRNAs were found to have a clear role in cancer. Of these filtered out 35 miRNAs, 15 miRNAs (42.8%) target NMT1 transcript, while 20 miRNAs (57%) target the NMT2 transcripts. These miRNAs are listed in Table 4. Furthermore, out of 35 miRNAs 14 (40%) miRNAs were found to be associated with prostate cancer whereas 4 (11.4%) miRNAs showed association with colorectal, breast and liver cancers. Additionally, 3/35 (8.5%) miRNAs were found to have roles in esophageal, renal and squamous cell carcinomas.

**Table 4:** The association of microRNAs (miRNAs) that target N-myristoyltransferase (*NMT*) transcript 1 and 2 (*NMT1* and *NMT2*) with different cancer types and their expression changes.

	*NMT1* targeting miRNAs	Specific	Up/Down Regulated	References	Reported functions
1	*miR-421*	Prostate Cancer	Down	[89]	repression of cancer cell proliferation and cell cycle
2	*miR-186-5p*	Prostate Cancer	Up	[90]	cell proliferation
3	*miR-675-5p*	Prostate Cancer	Down	[91]	metastasis
4	*miR-497-5p*	Prostate Cancer	Down	[92]	cell proliferation activity, migration and invasion
5	*miR-708-5p*	Prostate Cancer	Down	[93]	cell viability, migration, invasion, tumor progression, and reoccurrence
6	*miR-409-3p*	Colorectal Cancer	Down	[94]	metastasis
7	*miR-106b-3p*	Colorectal Cancer	Up	[95]	prognosis
8	*miR-140-5p*	Esophageal Cancer	Down	[96]	cell invasion
9	*miR-330-3p*	Esophageal Cancer	Down	[97]	cellular sensitivity to chemotherapy
10	*miR-376-5p*	Gastric Cancer	Up	[98]	cell growth
11	*miR-374b-5p*	Gastric Cancer	Up	[99]	cell invasion and metastasis
12	*miR-520b*	Liver Cancer	Down	[100]	tumorigenesis and liver colonization
13	*miR-127-3p*	Breast Cancer	Down	[101]	cell proliferation
14	*miR-503-5p*	Brain Cancer/Glioma	Down	[102]	cell proliferation invasion
15	*miR-346*	Squamous Cell Carcinoma	Up	[103]	cell proliferation and migration
	***NMT2* targeting miRNA**	**Specific**	**Up/Down Regulated**	**References**	**Reported functions**
1	*miR-376a-5p*	Prostate Cancer	Down	[104]	cell proliferation
2	*mIR-497*	Prostate Cancer	Down	[92]	cell proliferation activity, migration and invasion
3	*miR-1193*	Prostate Cancer	Down	[105]	cell proliferation, cell viability, and colony formation
4	*miR-195-5p*	Prostate Cancer	Down	[106]	migration and invasion
5	*miR-1205*	Prostate Cancer	Down	[107]	prostate cancer susceptibility
6	*miR-214-3p*	Prostate Cancer	Down	[108]	biomarker
7	*miR-1914-5p*	Colorectal Cancer	Upregulated	[109]	decreases chemo resistance abilities of CRC cells
8	*miR-421*	Prostate Cancer	Down	[89]	repression of cancer cell proliferation and cell cycle
9	*miR-212-5p*	Prostate Cancer	Down	[110]	angiogenesis and cellular senescence
10	*miR-301a-5p*	Colorectal Cancer	Up	[111]	migration and invasion
11	*miR-133a-5p*	Colorectal Cancer	Down	[112]	apoptosis and inhibiting cell proliferation
12	*miR-187-5p*	Breast Cancer	Up	[113]	poor prognosis, disease progression, poor outcome
13	*miR-411-5p*	Breast Cancer	Down	[114]	proliferation and metastasis
14	*miR-519e-5p*	Nasopharyngeal Cancer	Down	[115]	Inhibit upregulated gene-4
15	*miR-15b-5p*	Liver Cancer	Up	[116]	proliferation
16	*miR-199a-5p*	Liver Cancer	Down	[117]	low survival, higher tumor growth
17	*miR-548p*	Liver Cancer	Down	[118]	proliferation
18	*miR_200a-3p*	Renal Cancer	Down	[119]	cell proliferation, tumor suppressor
19	*miR-137*	Breast Cancer	Regulatory	[120]	Decreases expression of NOTCH
20	*miR-452-5p*	Esophageal Cancer	Up	[121]	molecular marker

#### Stem cell, T cell and B cell receptor signaling

A thorough search of the identified 221 miRNAs revealed a defined role for 21 (9.5%) miRNAs in TCR, BCR and stem cell signaling. Of the 21 miRNAs, 13 and 8 miRNAs contained complementarity with NMT1 and NMT2 transcripts’ 3’UTR, respectively. The differential regulation of miRNAs that are associated with signaling pathways are summarized in Table 3 and may provide insights on how differential expression of miRNAs may impact NMT levels.

### Infectious diseases

We found 14 miRNAs that were associated with functions in infectious diseases, 8 of which target *NMT1/2* genes and 6 miRNAs target *MetAP*2 gene. Of the total 14 miRNAs targeting either *NMT1/2* genes or *MetAP* gene, 13 miRNAs (*miR-29a-5p*, *miR-132-5p*, *miR-134-5p*, *miR-137*, *miR-139-3p*, *miR-140-5p*, *miR-199a-3p*, *mir-520d-5p*, *miR-548p*, *miR-943*, *miR-4317 and miR-628-3p*) have been found to have roles in viral infections and 4 miRNAs (*miR-127-3p*, *miR-140-5p and miR-199a-3p* and miR*-4317*) have been implicated in bacterial infections. Importantly, two miRNAs: *miR-199a-3p* and *miR-4317* play a role in both viral and bacterial infections. Furthermore, two miRNAs (*miR-132-5p* and *miR-140-5p*), have roles in both viral and fungal infections.

#### HIV infection

The *NMT1/2* targeting miR-137 and *MetAP2 targeting* miR-199a-3p were found to be upregulated in HIV infection, while another miRNA-324-5p, which targets *NMT1/2* genes, also modulates HIV infection by targeting viral infectivity factor gene.

#### Hepatitis virus infection

Four miRNAs were found to be associated with hepatitis viral infections namely *miR-29a-5p*, *miR-548p*, *miR-199a-3p* and *miR-520d-5p*. Of these four miRNAs, three miRNAs (*miR-29a-5p*, *miR-199a-3p*, *miR-520d-5p*) targeted *MetAP2* whereas, the fourth *miR-548p* targeted *NMT1/2* genes.

#### Human Papilloma Virus (HPV) infection

The two miRNAs (*miR-324-5p* and *miR-139-3p*) that were found to be associated with HPV infection also targeted *NMT1/2 and MetAP2 genes*. Human Papillomavirus subtype-16 E5 protein downregulates *miR-324-5p* and contrary to this miR-139-3p is downregulated in HPV-16-induced carcinomas.

#### Fungal infection

The *NMT1/2* and *MetAP2* genes’ targeting *miR-132-5p* was found to be associated with fungal infection of human dendritic cells with *Candida albicans* and *Aspergillus fumigatus* and regulates immune responses through the interactions with *FKBP1B*, *KLF4*, and *SPN genes*. Furth*ermore*, *miR-140*, *miR-628-3p* and *miR-943* miRNAs that target *NMT1/2* and *MetAP2* genes were found to have roles in infectious diseases. Of which miR-943 is upregulated in Herpes Zoster viral infection and miR-628-3p serves as a biomarker for the enterovirus 71 infection. The variable expression of miRNAs associated with infectious diseases is summarized in Table 5 with their corresponding references.

**Table 5:** The association of microRNAs (miRNAs) that target N-myristoyltransferase (*NMT*) transcript 1 and 2 (*NMT1* and *NMT2*) and *MetAP* transcripts with different infectious diseases and their expression changes.

	*NMT1/2 & MetAP miRNAs*	Specific	Up/Down Regulated	References
1	*miR-127-3p*	Bacterial infection	Up	[122]
2	*miR-132-5p*	Fungal infection with Candida albicans and Aspergillus fumigatus	Up	[123]
3	*miR-134-5p*	Poliovirus infection	Up	[124]
4	*miR-137*	HIV neurodegenerative diseases	down	[125]
5	*miR-140-5p*	Coxsackievirus/fungal infection	Up	[126, 127]
6	*miR-548p*	Suppresses HBV associated HCC	Down	[128]
7	*miR-943*	Herpes Zoster viral infection	Up	[129]
8	*miR-4317*	E Coli induced miRNA	Up	[130]
9	*miR-29a-5p*	HBV associated HCC	Up	[131]
10	*miR-139-3p*	HPV-16 induced Cancer	Down	[132]
11	*miR-199a-3p*	HBV/HCV/ Schistosoma mansoni	Regulatory	[133–138]
12	*miR-324-5p*	HCV/HIV/HPV and related cancers	Downregulated	[139–143]
13	*miR-520-5p*	Immune clearance of HBV	Regulatory	[142]
14	*miR-628-3p*	Biomarker for enterovirus 71 infection	Diagnostic	[126]

### Targeting miRNAs of NMT follows RNA Regulon model

**Fig 6:**
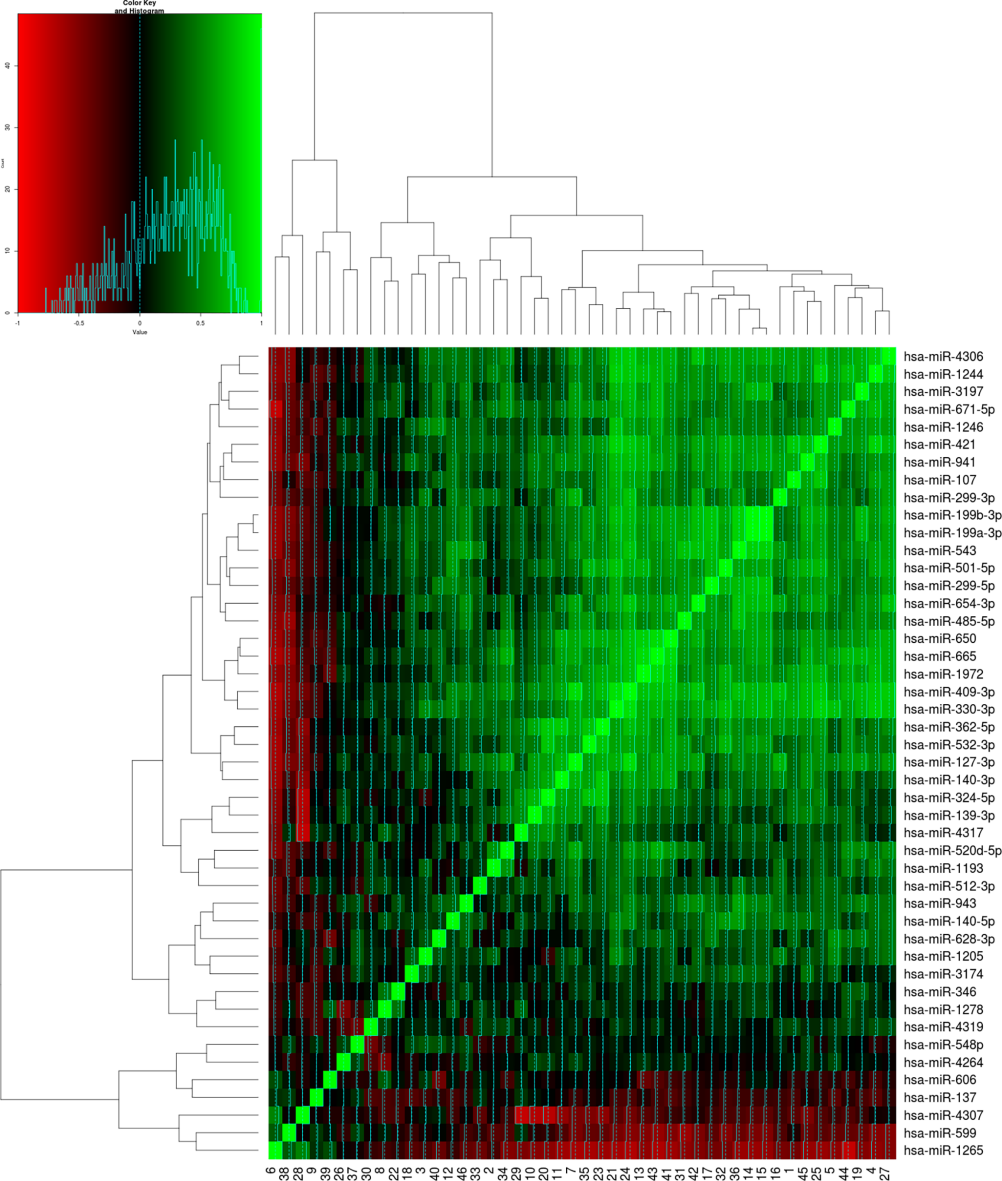
The clustering of microRNAs (miRNAs) targeting N-myristoyltransferase *(NMT)* genes. The figure illustrates clustering of the miRNAs that target *NMT1/2* genes based on normalized expression values obtained from “GSE62037”. Data suggest miRNA regulon model is similar to the RNA regulon model. Green color shows positive Pearson correlation coefficient (PCC) values (0.5 ≤ *r* ≤ 1) and red color shows inverse/negative PCC values (-0.5 ≤ *r* ≤ 1). Heatmap shows that most of miRNAs have high PCC values, and these miRNAs co-express with each other to regulate common biological processes.

The miRNAs targeting *NMT1/2* genes were clustered together based on their expression, which is supported by a previous report proposing that miRNAs performing similar functions usually co-express with each other [61]. This phenomenon of miRNA regulon was previously observed between miRNAs regulated by same RNA Binding Proteins [144]. We deemed it worth to identify whether *NMT1/2* and *MetAP2* gene expression followed a pattern indicative of a miRNA regulon. To find an active regulon model, PCC between miRNAs were calculated based on the expression values and miRNAs were clustered based on the correlation value. It was observed that *miR-4306* share highest co-expression with *miR-1244* followed by *miR-3179* (Fig 6). This regulon model suggests that some common biological pathway is regulated by these miRNAs apart from targeting of *NMT1/2* genes. Our data supports this notion as many of the miRNAs detected in this study were found to be involved in targeting genes involved in same pathways. Most of these miRNAs co-express with each other indicating miRNA regulon model is similar to the RNA regulon model, which needs further evaluation.

## Discussion

Understanding miRNA-mRNA interactions that play role in tumor development, cancer progression and associated cellular processes such as immune responses is a critical step necessary to use miRNAs as therapeutic or diagnostic targets for cancer and other diseases. miRNAs are emerging as a crucial component in the regulation of cellular signaling pathways, including several processes involved in diseases such as cancer and immune dysfunction. Investigating miRNA function and expression patterns is necessary for fully understanding how miRNAs fit to interact with more well studied mechanisms of cell regulation, giving us a bigger and clearer picture of the signaling framework of cells under various conditions. Specifically, studying miRNA interactions with the *NMT1/2* and *MetAP2* genes will shed light on the poorly understood regulation of myristoylation, a key cellular function critical to oncogenesis, adaptive immune function, and infectious disease onset. Our findings have verified that miRNAs are likely an important driving force in the regulation of myristoylation, and identified several NMT-transcript-binding miRNAs that have been linked to processes mediated by myristoylation.

The present study predicted (1) miRNAs that significantly bind to the NMT1 and NMT2 transcripts, (2) miRNAs in which NMT1/2 and MetAP2 transcripts are common binding targets, (3) a possible role for the involvement of NMT1/2 transcript-binding miRNAs in Cancer, T/B cell receptor signaling and infectious diseases. By using TargetScan software, we initially found a total of 13,798 miRNA-target hits for the *NMT1/2* genes, whereas the *MetAP2* gene had 7,708 interactions detected. Statistical analysis using “R” software package further delineated significant binding of these miRNAs onto their target; based on this, 221 NMT miRNA-NMT-target interactions, and 165 miRNA-METAP2-target interactions were verified. A thorough literature review was performed to find if these miRNAs have any role in Cancer, T/B cell receptor signaling, and infectious diseases. Of the 35 miRNAs, 15 (42.8%) target the NMT1 transcript and 20 (57%) target the NMT2 transcripts. Interestingly, 14 miRNAs (40%) have a defined role in prostate cancer, and 4 (11.4%) miRNAs each that play a role in colorectal, breast and liver cancers. Furthermore 3 (8.5%) miRNAs are involved in esophageal cancer, and one miRNA each that has been detected to play a role in renal cell carcinoma, brain cancer, and role in squamous cell carcinomas. Secondly, with regards to T/B cell receptor signaling, 21 (9.5%) *NMT1/2* targeting miRNAs were detected. Of the 21 miRNAs, 13 had *NMT1* as their target site and 8 had *NMT2* as their target site. Lastly, 2 of each of the miRNAs targeting *NMT1/2* were involved in infectious diseases, including HIV.

Numerous recent reports clearly point to a role of miRNA in cancer establishment, evasion, differentiation, and metastasis, which when targeted, have shown the promising results as therapeutic targets in preclinical as well as in clinical trials [145, 146]. Our findings indicate that several miRNAs that target myristoylation related genes (*NMT1/*2 and M*etAP2*) are associated with cancer. Interestingly, of these identified miRNAs, some have been found to be associated with colorectal or breast cancers wherein role of NMT has already been established [2, 50]. Furthermore, miR-409-3p and miR-106b-3p, and miR-127 that all bind to the *NMT1* transcript, have been reportedly involved in colorectal cancer and breast cancer respectively. miR-409-3p was previously found to be a colorectal tumor suppressor, with reduced expression of miR-409p present in colorectal cancer tissue and correlated with metastasis [94]. In contrast, miR-106b-3p upregulation was correlated with colorectal tumor growth [147]. Wang et al demonstrated that miR-127 is downregulated in breast cancer tissues, and reduced miR-127 expression is correlated with late stage lymph node metastasis. Furthermore, miR-127 downregulation was an independent prognostic factor that predicted lower overall survival in breast cancer patients and upregulation of miR-127 inhibited breast cancer cells growth, survival, and migration [148].

We also identified NMT2-targeting miRNAs that have been previously demonstrated to play a role in regulating or advancing colorectal and breast cancers. Our analysis identified miR-133a and miR-301a as NMT2-binding miRNAs that act to suppress and promote colorectal cancer respectively. Wan et al established through gene analysis that miR-133a expression was reduced in 83.2% of colorectal cancer patient tumors compared to healthy mucosal tissue, and Dong et al demonstrated that ectopic overexpression of miR-133a suppresses colorectal tumor growth in-vitro and in-vivo [112, 149]. A study into the colorectal cancer promoting mechanism of miR-301a, which is upregulated in colorectal cancers, revealed that miR-301a expression confers growth and invasion by downregulating SOCS6 expression [150]. Our investigation of NMT2 transcript-binding miRNAs revealed miR-411-5p and miR-187, which respectively function as a tumor suppressor and an oncogenic agent of breast cancer. miR-411-5p was observed to suppress breast cancer by downregulating GRB2 and Ras expression [114]. Finally, a report by Mulrane et al identified miR-187 expression as an independent factor that drives the in vitro development of increased aggressiveness and invasiveness of breast cancer [113].

There are several lines of evidence that demonstrate the critical role miRNAs play in stem cell, T-cell, and B-cell signaling [151–154]. This is of relevance to this study as besides cancer, studies on myristoylation signaling have focused on NMT’s role with respect to embryogenesis, innate immune cell differentiation, and adaptive immune development and signaling, and HIV infection [4, 5, 8, 10]. During our analysis we identified several NMT1/2-binding-miRNAs involved in the previously mentioned functions and have chosen to discuss a selection of the most relevant and best-annotated examples. With regards to certain developmental pathways, miR-200, 199b and 26a have been shown to play a decisive role, with the former binding to the NMT1 transcript, and latter two binding to the NMT2 transcript. miR-200 family microRNAs were found to be expressed exclusively in epithelial type tissues and when downregulated could stimulate an epithelial-mesenchymal transition, which may implicate their reduced activity with cancer metastasis [155]. miR-199b has been shown to block stem cell differentiation through inhibition of the Notch pathway [81]. miR-26a was identified as a required factor for the differentiation of skeletal muscle during mouse development, with inhibition of miR-26a resulting in delayed muscle regeneration [156].

In terms of immune function, the NMT2-transcript binding miR-628 was found to be upregulated following TLR mediated LPS detection in monocytes, and targeted MyD88 as part of a negative feedback loop for TLR signaling [157]. Two NMT1-transcript-binding miRNAs, miR-29b and 326, have evident roles in T-cell signaling. miR-29b overexpression in CD8+ T cells of renal carcinoma patients has been found to induce immune dysfunction through down-regulation of JAK3 and MCL-1 [158]. Furthermore, heightened expression of miR-326, which is associated with multiple sclerosis (MS), was shown to drive TH-17 T helper cell differentiation and MS pathogenesis in mouse models [78]. Two mRNA hits during our analysis were functionally implicated in the regulation of B-cell signaling, specifically with regards to the autoimmune disease lupus. The B-cells of lupus patients were determined to have increased expression of miR-148, an NMT1-transcript-binding miRNA, and decreased expression of miR-1246, an NMT2-transcript-binding miRNA. miR-148a is thought to affect B cell signaling by impairing immune tolerance pathways through suppression of PTEN, thus contributing to autoimmune symptoms [159]. In contrast, expression of miR-1246 normally regulated B cell activation through suppression of EBF1, however miR-1246 expression is reduced in the activated B cells of lupus patients through Akt-p53 signaling [160]. Lastly, miR-132 (NMT1-transcript-binding miRNA) and miR-29 (NMT2-transcript-binding miRNA) are involved in HIV infection, with the former promoting infection and the latter acting as an antiviral agent. miR-132 is highly upregulated during CD4+ T cell activation and are thought to enhance HIV-1 replication [161]; miR-29 family miRNAs are expressed by increased STAT3 transcription following IL-21 stimulation and lead to an anti-viral environment in CD4+ T cells during initial control of HIV-1 in vivo [162].

The overlap between the effects of NMT-transcript-binding miRNAs and previously reported NMT dysfunction are encouraging for the future development of therapeutics or diagnostic/prognostic biomarkers that exploit myristoylation-mediated cell signaling. The high stability of miRNAs have made them a promising biomarkers as they can be easily obtained from the buffy coat associated immune cells of blood samples [163, 164]. Additionally, methods of in vivo miRNA modulation are being developed for future clinical settings [165].

A recent report by Chen et al. documented dynamic interaction of *miR-99* and *miR-100* and the ability of *miR-100* to downregulate *NMT1* transcript levels [166]. Several other recent studies demonstrated that *miR-99* modulates many molecular signaling pathways that are not limited to AKT, mTOR, MMP1 and IGFR1 signaling and contribute to the tumorigenesis of cancer conditions such as head and neck squamous cell carcinoma [167], oral squamous cell carcinoma [168, 169] and esophageal squamous cell carcinoma [169]. Interestingly, one functional study indeed proved that *miR-99a* inhibits cell proliferation, colony formation ability, migration and invasion by targeting fibroblast growth factor receptor 3 (*FGFR3*) in prostate cancer [170] and bladder cancer [171]. A study by Androulidaki et al. demonstrated that AKT1 controls macrophage responses to the LPS by regulation miRNA [172]. All these studies provide supporting evidence that *miR-99* is a critical player in cancer pathogenesis, which is possibly derived from post-transcriptional regulation of *NMT*. Study by Schramedei et al. demonstrated that *MetAP2* is a putative target *of miR-21* in B-cell lymphomas [173].

In addition to prediction of the nature of miRNA-mRNA relationships, our study also revealed another layer of complexity and interconnectedness of miRNAs, long noncoding RNAs (lncRNA) and target mRNAs. The *NMT1* targeting *miR-675-5p*, which is down regulated in metastatic prostate cancer, derives from the lncRNA *H19* [91]. In prostate cancer cells, *H19* is upregulated that aids in expression of *miR-675*. The expression of lncRNA H19 and *miR-675* were associated with repression of extracellular matrix protein, TGFβ1, that regulate cellular migration and cancer metastasis. It appears that the *H19-miR-675* lncRNA-miRNA interactions function in different cancer cell types by targeting different mRNAs. For instance, in gastric cancer, *H19-miR-675* regulates cell proliferation by repressing *RUNX1* [174].

One of the bottlenecks in HIV infection is the neurodegeneration, which occurs through modulation of host axon guidance and associated neurotrophin signaling pathways. Zhou et al., comprehensively profiled miRNAs in patients with dementia who were infected with HIV. In this study, they uncovered three important miRNAs that were dysregulated particularly in HIV infected dementia patients compared to only dementia patients. The *miR-137* that targets *NMT1/2* and *MetAP2* genes was on top of the list. Further enrichment of the miRNA pathways revealed that *miR-137* is involved in more than 5 neurodegeneration pathways that included WNT and MAPK pathways, which happens to be dysregulated in cancers as well [125]. Since miR-137 targets *NMT1/2* genes and particularly in dentate gyrus and hippocampus, it would be interesting to further investigate the dynamics of miR-137 and NMT in dementia patients with HIV. Hepatocellular carcinoma is a dreaded cancer of the liver whose etiology is linked to chronic liver inflammation most commonly due to infection with hepatitis B and hepatitis C viruses. Our analysis revealed four *NMT1/2* and *MetAP2* targeting miRNAs (miR-29a-5p, miR-548p, miR-199a-3p and miR-520d-5p) that have roles in hepatitis viral infections. miR-29a-5p is demonstrated to be upregulated in Hepatitis B Virus infection related to hepatocellular carcinoma and may function through inhibition of PTEN (131). Mounting evidences in the literature suggest that miR-199 plays dominant and multi-faceted role not only in cancer but also in infectious diseases including HBV, HCV and Schistosoma mansoni. It not only inhibits the HBV viral replication but also regresses the hepatocellular carcinoma [47, 133]. Moreover, miR-199a-3p can also be used as early biomarker in HCV infection [138]. As revealed in our analysis, since miR-199a-3p also inhibits the *MetAP2* gene, which plays key role in angiogenesis, further studies are warranted to underpin the role of miR-199a-3p in oncogenesis. Apart from these miRNAs, we also found that *miR-520d-5p* is associated with immune clearance by transitioning the immune tolerant to immune activation state in chronic hepatitis B and *miR-548p* suppresses hepatitis B virus associated HCC by downregulating expression of hepatitis B x-interacting protein [128, 175].

Our in-depth analyses of miRNAs that regulate NM*T1/2* and *MetAP2* genes have cemented the significance of miRNAs in disease etiology and progression. The emergence of miRNAs’ abilities in regulating various important facets of cellular functions dictates the significance of miRNAs in designing new therapies around cell signaling molecules.

Several miRNAs in this study were predicted to interact with mRNA transcripts responsible for translating the key proteins involved in myristoylation (NMT1/NMT2/METAP2). This preliminary *in-silico* analysis requires future validation within in-vitro cell models to demonstrate expression of the identified miRNAs and their interaction with the target transcripts.

Initial validation should include quantitative PCR (qPCR) to confirm co-expression of each miRNA and their respective transcript target using miRNA and transcript specific primers. Since, *in-silico* study may reveal a large number of potential miRNA binding sites on a target mRNA, it is important that a reporter assay should be performed to validate specific binding sites.

The interaction between a miRNA of interest and its target(s) needs to be demonstrated, which can be done using in-situ hybridization with locked nucleic acid (LNA) modified oligonucleotide probes labelled with digoxigenin that can be visualized appropriately using digoxigenin antibody conjugated to alkaline phosphatase to act upon a chromogenic substrate. A major challenge to these initial approaches includes a potential tissue specific expression of the analyzed miRNAs, emphasizing the need to test a spectrum of tissue types and cell lines. Tissue specific expression of NMT1, NMT2 and MetAP2 is well documented and therefore, initial studies would require validation of miRNAs in cell lines and tissues that express the target genes.

Furthermore, miRNAs of interest may be overexpressed (gain-in-function) in cell lines through a lentiviral infection or plasmid transfection to determine their effect on NMT1/NMT2/METAP2 mRNA transcript levels using qPCR and the resulting protein expression by Western blot analysis. Narrowed down miRNAs from the aforementioned validation techniques will be used for further studies in order to determine their effect(s) on cell signaling pathways that are involved in regulating cell proliferation, apoptosis, T/B cell signaling and viral infection.

## Conclusion

Our original data set encompassed over 13, 000 miRNAs that potentially target and regulate NMT. Together, the functional data available for NMT1/2-transcript-targeting miRNAs helped create a condensed data set of miRNAs that serve as strong candidates for further investigation into the regulation of myristoylation with respect to various types of signaling dysregulation. In total, 35 NMT-transcript-binding miRNAs were linked to cancer, 21 linked to development or immune dysfunction, and 14 linked to infectious disease, including HIV and HBV infection. Future analysis of this miRNA panel is needed to verify a direct link between these miRNAs and myristoylation dysfunction associated diseases. This study provides a platform for studying an additional facet of NMT regulation, and may lead to the development of new therapeutic targets or biomarkers for several types of cancer, development or immune dysfunction, HIV and hepatitis viral infections.

## Supporting information

S1 Table Putative miRNAs that target N-myristoyltransferase (NMT) gene. (DOCX)

S2 Table Putative microRNAs (miRNAs) that target methionine aminopeptidase 2 *(METAP2)* gene. (DOC)

## Abbreviations

NMT: *N*-Myristoyltransferase
METAP2: Methionine aminopeptidase 2
miRNAs: microRNAs
ncRNAs: noncoding RNAs
lncRNAs: long noncoding RNAs
UTR: untranslated region
Infernal: INFERence of RNA Alignment
NCBI: National Center for Biotechnology Information
GEO: Gene Expression Omnibus
PCC: Pearson correlation-coefficient
GO: Gene Ontology
KEGG: Kyoto Encyclopedia of Genes and Genomes
*AKT*: Protein kinase B
*GSK3B*: Glycogen synthase kinase 3 beta
*BRAF*: B-Raf proto-oncogene
*MAPK*: Mitogen-activated protein kinase
HBV: Hepatitis B virus
HCC: hepatocellular carcinoma
