# Supplementary material for: In silico identification of microRNAs predicted to regulate N-myristoyltransferase and Methionine Aminopeptidase 2 functions in cancer and infectious diseases
Source: PLoS One. 2018 Mar 26;13(3):e0194612. doi: 10.1371/journal.pone.0194612 (PMC5868815; doi:10.1371/journal.pone.0194612)
Supplement: S1 Table — (DOCX) [file pone.0194612.s001.docx]

**S1 Table. Putative miRNAs that target N-myristoyltransferase (NMT) gene**

|  | **Gene**  **Name** | **Ensemble Transcript** | **Targeting miRNA** | **Start position** | **End position** | **Sequence of miRNA** | **PCC** | **P-value** |
| --- | --- | --- | --- | --- | --- | --- | --- | --- |
| 1 | *NMT* | ENST00000378165 | *miR-4317* | 370 | 375 | ACAUUGCCAGGGAGUUU | -0.673075357 | 4.59E-005 |
| 2 | *NMT* | ENST00000478580 | *miR-4317* | 410 | 415 | ACAUUGCCAGGGAGUUU | -0.673075357 | 4.59E-005 |
| 3 | *NMT* | ENST00000378150 | *miR-4317* | 348 | 353 | ACAUUGCCAGGGAGUUU | -0.673075357 | 4.59E-005 |
| 4 | *NMT* | ENST00000466201 | *miR-4317* | 249 | 254 | ACAUUGCCAGGGAGUUU | -0.673075357 | 4.59E-005 |
| 5 | *NMT* | ENST00000258960 | *miR-4317* | 2207 | 2212 | ACAUUGCCAGGGAGUUU | -0.673075357 | 4.59E-005 |
| 6 | *NMT* | ENST00000592782 | *miR-4317* | 2320 | 2325 | ACAUUGCCAGGGAGUUU | -0.673075357 | 4.59E-005 |
| 7 | *NMT* | ENST00000543908 | *miR-606* | 823 | 828 | AAACUACUGAAAAUCAAAGAU | -0.627277623 | 2.07E-004 |
| 8 | *NMT* | ENST00000378165 | *miR-421* | 1775 | 1781 | AUCAACAGACAUUAAUUGGGCGC | -0.596559775 | 5.02E-004 |
| 9 | *NMT* | ENST00000486786 | *miR-421* | 356 | 361 | AUCAACAGACAUUAAUUGGGCGC | -0.596559775 | 5.02E-004 |
| 10 | *NMT* | ENST00000590310 | *miR-421* | 634 | 641 | AUCAACAGACAUUAAUUGGGCGC | -0.596559775 | 5.02E-004 |
| 11 | *NMT* | ENST00000478580 | *miR-421* | 43 | 49 | AUCAACAGACAUUAAUUGGGCGC | -0.596559775 | 5.02E-004 |
| 12 | *NMT* | ENST00000378150 | *miR-421* | 1753 | 1759 | AUCAACAGACAUUAAUUGGGCGC | -0.596559775 | 5.02E-004 |
| 13 | *NMT* | ENST00000587120 | *miR-421* | 349 | 355 | AUCAACAGACAUUAAUUGGGCGC | -0.596559775 | 5.02E-004 |
| 14 | *NMT* | ENST00000258960 | *miR-421* | 4662 | 4668 | AUCAACAGACAUUAAUUGGGCGC | -0.596559775 | 5.02E-004 |
| 15 | *NMT* | ENST00000592782 | *miR-421* | 4775 | 4781 | AUCAACAGACAUUAAUUGGGCGC | -0.596559775 | 5.02E-004 |
| 16 | *NMT* | ENST00000591931 | *miR-140-5p* | 79 | 84 | CAGUGGUUUUACCCUAUGGUAG | -0.589396352 | 6.10E-004 |
| 17 | *NMT* | ENST00000590310 | *miR-941* | 410 | 415 | CACCCGGCUGUGUGCACAUGUGC | -0.578546168 | 8.11E-004 |
| 18 | *NMT* | ENST00000378165 | *miR-409-3p* | 1356 | 1361 | GAAUGUUGCUCGGUGAACCCCU | -0.575028328 | 8.88E-004 |
| 19 | *NMT* | ENST00000378165 | *miR-409-3p* | 2381 | 2387 | GAAUGUUGCUCGGUGAACCCCU | -0.575028328 | 8.88E-004 |
| 20 | *NMT* | ENST00000378165 | *miR-409-3p* | 2650 | 2656 | GAAUGUUGCUCGGUGAACCCCU | -0.575028328 | 8.88E-004 |
| 21 | *NMT* | ENST00000486786 | *miR-409-3p* | 148 | 153 | GAAUGUUGCUCGGUGAACCCCU | -0.575028328 | 8.88E-004 |
| 22 | *NMT* | ENST00000585561 | *miR-409-3p* | 649 | 654 | GAAUGUUGCUCGGUGAACCCCU | -0.575028328 | 8.88E-004 |
| 23 | *NMT* | ENST00000378150 | *miR-409-3p* | 1334 | 1339 | GAAUGUUGCUCGGUGAACCCCU | -0.575028328 | 8.88E-004 |
| 24 | *NMT* | ENST00000378150 | *miR-409-3p* | 2359 | 2365 | GAAUGUUGCUCGGUGAACCCCU | -0.575028328 | 8.88E-004 |
| 25 | *NMT* | ENST00000588455 | *miR-409-3p* | 224 | 229 | GAAUGUUGCUCGGUGAACCCCU | -0.575028328 | 8.88E-004 |
| 26 | *NMT* | ENST00000590114 | *miR-409-3p* | 551 | 556 | GAAUGUUGCUCGGUGAACCCCU | -0.575028328 | 8.88E-004 |
| 27 | *NMT* | ENST00000258960 | *miR-409-3p* | 573 | 578 | GAAUGUUGCUCGGUGAACCCCU | -0.575028328 | 8.88E-004 |
| 28 | *NMT* | ENST00000543908 | *miR-409-3p* | 446 | 451 | GAAUGUUGCUCGGUGAACCCCU | -0.575028328 | 8.88E-004 |
| 29 | *NMT* | ENST00000592782 | *miR-409-3p* | 686 | 691 | GAAUGUUGCUCGGUGAACCCCU | -0.575028328 | 8.88E-004 |
| 30 | *NMT* | ENST00000378165 | *miR-4307* | 2190 | 2195 | AAUGUUUUUUCCUGUUUCC | -0.571249582 | 9.77E-004 |
| 31 | *NMT* | ENST00000378165 | *miR-4307* | 780 | 785 | AAUGUUUUUUCCUGUUUCC | -0.571249582 | 9.77E-004 |
| 32 | *NMT* | ENST00000590310 | *miR-4307* | 97 | 102 | AAUGUUUUUUCCUGUUUCC | -0.571249582 | 9.77E-004 |
| 33 | *NMT* | ENST00000478580 | *miR-4307* | 820 | 825 | AAUGUUUUUUCCUGUUUCC | -0.571249582 | 9.77E-004 |
| 34 | *NMT* | ENST00000585561 | *miR-4307* | 787 | 792 | AAUGUUUUUUCCUGUUUCC | -0.571249582 | 9.77E-004 |
| 35 | *NMT* | ENST00000378150 | *miR-4307* | 2168 | 2173 | AAUGUUUUUUCCUGUUUCC | -0.571249582 | 9.77E-004 |
| 36 | *NMT* | ENST00000378150 | *miR-4307* | 758 | 763 | AAUGUUUUUUCCUGUUUCC | -0.571249582 | 9.77E-004 |
| 37 | *NMT* | ENST00000588455 | *miR-4307* | 362 | 367 | AAUGUUUUUUCCUGUUUCC | -0.571249582 | 9.77E-004 |
| 38 | *NMT* | ENST00000592654 | *miR-4307* | 177 | 183 | AAUGUUUUUUCCUGUUUCC | -0.571249582 | 9.77E-004 |
| 39 | *NMT* | ENST00000258960 | *miR-4307* | 711 | 716 | AAUGUUUUUUCCUGUUUCC | -0.571249582 | 9.77E-004 |
| 40 | *NMT* | ENST00000543908 | *miR-4307* | 584 | 589 | AAUGUUUUUUCCUGUUUCC | -0.571249582 | 9.77E-004 |
| 41 | *NMT* | ENST00000592782 | *miR-4307* | 824 | 829 | AAUGUUUUUUCCUGUUUCC | -0.571249582 | 9.77E-004 |
| 42 | *NMT* | ENST00000591931 | *miR-1265* | 630 | 636 | CAGGAUGUGGUCAAGUGUUGUU | -0.566257251 | 0.001106752 |
| 43 | *NMT* | ENST00000378165 | *miR-1265* | 1404 | 1409 | CAGGAUGUGGUCAAGUGUUGUU | -0.566257251 | 0.001106752 |
| 44 | *NMT* | ENST00000378165 | *miR-1265* | 1762 | 1767 | CAGGAUGUGGUCAAGUGUUGUU | -0.566257251 | 0.001106752 |
| 45 | *NMT* | ENST00000486786 | *miR-1265* | 196 | 201 | CAGGAUGUGGUCAAGUGUUGUU | -0.566257251 | 0.001106752 |
| 46 | *NMT* | ENST00000378150 | *miR-1265* | 1382 | 1387 | CAGGAUGUGGUCAAGUGUUGUU | -0.566257251 | 1.11E-003 |
| 47 | *NMT* | ENST00000378150 | *miR-1265* | 1740 | 1745 | CAGGAUGUGGUCAAGUGUUGUU | -0.566257251 | 1.11E-003 |
| 48 | *NMT* | ENST00000587120 | *miR-1265* | 225 | 231 | CAGGAUGUGGUCAAGUGUUGUU | -0.566257251 | 0.001106752 |
| 49 | *NMT* | ENST00000587670 | *miR-1265* | 104 | 110 | CAGGAUGUGGUCAAGUGUUGUU | -0.566257251 | 0.001106752 |
| 50 | *NMT* | ENST00000258960 | *miR-1265* | 1738 | 1744 | CAGGAUGUGGUCAAGUGUUGUU | -0.566257251 | 0.001106752 |
| 51 | *NMT* | ENST00000258960 | *miR-1265* | 2286 | 2291 | CAGGAUGUGGUCAAGUGUUGUU | -0.566257251 | 0.001106752 |
| 52 | *NMT* | ENST00000592782 | *miR-1265* | 1851 | 1857 | CAGGAUGUGGUCAAGUGUUGUU | -0.566257251 | 0.001106752 |
| 53 | *NMT* | ENST00000592782 | *miR-1265* | 2399 | 2404 | CAGGAUGUGGUCAAGUGUUGUU | -0.566257251 | 0.001106752 |
| 54 | *NMT* | ENST00000378165 | *miR-665* | 2033 | 2038 | ACCAGGAGGCUGAGGCCCCU | -0.56224085 | 1.22E-003 |
| 55 | *NMT* | ENST00000378165 | *miR-665* | 2139 | 2144 | ACCAGGAGGCUGAGGCCCCU | -0.56224085 | 1.22E-003 |
| 56 | *NMT* | ENST00000378165 | *miR-665* | 973 | 978 | ACCAGGAGGCUGAGGCCCCU | -0.56224085 | 0.001222009 |
| 57 | *NMT* | ENST00000590310 | *miR-665* | 499 | 506 | ACCAGGAGGCUGAGGCCCCU | -0.56224085 | 1.22E-003 |
| 58 | *NMT* | ENST00000378150 | *miR-665* | 2011 | 2016 | ACCAGGAGGCUGAGGCCCCU | -0.56224085 | 0.001222009 |
| 59 | *NMT* | ENST00000378150 | *miR-665* | 2117 | 2122 | ACCAGGAGGCUGAGGCCCCU | -0.56224085 | 0.001222009 |
| 60 | *NMT* | ENST00000378150 | *miR-665* | 951 | 956 | ACCAGGAGGCUGAGGCCCCU | -0.56224085 | 0.001222009 |
| 61 | *NMT* | ENST00000543908 | *miR-665* | 1042 | 1047 | ACCAGGAGGCUGAGGCCCCU | -0.56224085 | 0.001222009 |
| 62 | *NMT* | ENST00000378165 | *miR-1193* | 770 | 775 | GGGAUGGUAGACCGGUGACGUGC | -0.560977445 | 0.001260365 |
| 63 | *NMT* | ENST00000590310 | *miR-1193* | 87 | 92 | GGGAUGGUAGACCGGUGACGUGC | -0.560977445 | 0.001260365 |
| 64 | *NMT* | ENST00000478580 | *miR-1193* | 810 | 815 | GGGAUGGUAGACCGGUGACGUGC | -0.560977445 | 0.001260365 |
| 65 | *NMT* | ENST00000585561 | *miR-1193* | 777 | 782 | GGGAUGGUAGACCGGUGACGUGC | -0.560977445 | 0.001260365 |
| 66 | *NMT* | ENST00000378150 | *miR-1193* | 748 | 753 | GGGAUGGUAGACCGGUGACGUGC | -0.560977445 | 0.001260365 |
| 67 | *NMT* | ENST00000588455 | *miR-1193* | 352 | 357 | GGGAUGGUAGACCGGUGACGUGC | -0.560977445 | 0.001260365 |
| 68 | *NMT* | ENST00000587670 | *miR-1193* | 430 | 436 | GGGAUGGUAGACCGGUGACGUGC | -0.560977445 | 0.001260365 |
| 69 | *NMT* | ENST00000587014 | *miR-1193* | 221 | 228 | GGGAUGGUAGACCGGUGACGUGC | -0.560977445 | 0.001260365 |
| 70 | *NMT* | ENST00000258960 | *miR-1193* | 1244 | 1251 | GGGAUGGUAGACCGGUGACGUGC | -0.560977445 | 0.001260365 |
| 71 | *NMT* | ENST00000258960 | *miR-1193* | 2255 | 2260 | GGGAUGGUAGACCGGUGACGUGC | -0.560977445 | 0.001260365 |
| 72 | *NMT* | ENST00000258960 | *miR-1193* | 3897 | 3903 | GGGAUGGUAGACCGGUGACGUGC | -0.560977445 | 1.26E-003 |
| 73 | *NMT* | ENST00000258960 | *miR-1193* | 701 | 706 | GGGAUGGUAGACCGGUGACGUGC | -0.560977445 | 0.001260365 |
| 74 | *NMT* | ENST00000543908 | *miR-1193* | 574 | 579 | GGGAUGGUAGACCGGUGACGUGC | -0.560977445 | 0.001260365 |
| 75 | *NMT* | ENST00000592782 | *miR-1193* | 1357 | 1364 | GGGAUGGUAGACCGGUGACGUGC | -0.560977445 | 0.001260365 |
| 76 | *NMT* | ENST00000592782 | *miR-1193* | 2368 | 2373 | GGGAUGGUAGACCGGUGACGUGC | -0.560977445 | 0.001260365 |
| 77 | *NMT* | ENST00000592782 | *miR-1193* | 4010 | 4016 | GGGAUGGUAGACCGGUGACGUGC | -0.560977445 | 0.001260365 |
| 78 | *NMT* | ENST00000592782 | *miR-1193* | 814 | 819 | GGGAUGGUAGACCGGUGACGUGC | -0.560977445 | 0.001260365 |
| 79 | *NMT* | ENST00000591931 | *miR-943* | 403 | 408 | CUGACUGUUGCCGUCCUCCAG | -0.560732846 | 0.001267911 |
| 80 | *NMT* | ENST00000258960 | *miR-943* | 1511 | 1516 | CUGACUGUUGCCGUCCUCCAG | -0.560732846 | 0.001267911 |
| 81 | *NMT* | ENST00000592782 | *miR-943* | 1624 | 1629 | CUGACUGUUGCCGUCCUCCAG | -0.560732846 | 1.27E-003 |
| 82 | *NMT* | ENST00000591931 | *miR-1205* | 329 | 335 | UCUGCAGGGUUUGCUUUGAG | -0.549608986 | 0.001655472 |
| 83 | *NMT* | ENST00000378165 | *miR-1205* | 708 | 714 | UCUGCAGGGUUUGCUUUGAG | -0.549608986 | 0.001655472 |
| 84 | *NMT* | ENST00000590310 | *miR-1205* | 282 | 288 | UCUGCAGGGUUUGCUUUGAG | -0.549608986 | 0.001655472 |
| 85 | *NMT* | ENST00000478580 | *miR-1205* | 748 | 754 | UCUGCAGGGUUUGCUUUGAG | -0.549608986 | 0.001655472 |
| 86 | *NMT* | ENST00000585561 | *miR-1205* | 83 | 88 | UCUGCAGGGUUUGCUUUGAG | -0.549608986 | 1.66E-003 |
| 87 | *NMT* | ENST00000378150 | *miR-1205* | 686 | 692 | UCUGCAGGGUUUGCUUUGAG | -0.549608986 | 1.66E-003 |
| 88 | *NMT* | ENST00000588455 | *miR-1205* | 547 | 553 | UCUGCAGGGUUUGCUUUGAG | -0.549608986 | 0.001655472 |
| 89 | *NMT* | ENST00000590114 | *miR-1205* | 83 | 88 | UCUGCAGGGUUUGCUUUGAG | -0.549608986 | 1.66E-003 |
| 90 | *NMT* | ENST00000466201 | *miR-1205* | 487 | 492 | UCUGCAGGGUUUGCUUUGAG | -0.549608986 | 0.001655472 |
| 91 | *NMT* | ENST00000587670 | *miR-1205* | 464 | 470 | UCUGCAGGGUUUGCUUUGAG | -0.549608986 | 0.001655472 |
| 92 | *NMT* | ENST00000588975 | *miR-1205* | 111 | 116 | UCUGCAGGGUUUGCUUUGAG | -0.549608986 | 0.001655472 |
| 93 | *NMT* | ENST00000258960 | *miR-1205* | 105 | 110 | UCUGCAGGGUUUGCUUUGAG | -0.549608986 | 0.001655472 |
| 94 | *NMT* | ENST00000258960 | *miR-1205* | 1437 | 1443 | UCUGCAGGGUUUGCUUUGAG | -0.549608986 | 0.001655472 |
| 95 | *NMT* | ENST00000258960 | *miR-1205* | 2171 | 2177 | UCUGCAGGGUUUGCUUUGAG | -0.549608986 | 0.001655472 |
| 96 | *NMT* | ENST00000258960 | *miR-1205* | 3103 | 3109 | UCUGCAGGGUUUGCUUUGAG | -0.549608986 | 0.001655472 |
| 97 | *NMT* | ENST00000258960 | *miR-1205* | 3179 | 3185 | UCUGCAGGGUUUGCUUUGAG | -0.549608986 | 0.001655472 |
| 98 | *NMT* | ENST00000258960 | *miR-1205* | 3233 | 3239 | UCUGCAGGGUUUGCUUUGAG | -0.549608986 | 0.001655472 |
| 99 | *NMT* | ENST00000258960 | *miR-1205* | 3931 | 3937 | UCUGCAGGGUUUGCUUUGAG | -0.549608986 | 1.66E-003 |
| 100 | *NMT* | ENST00000258960 | *miR-1205* | 896 | 902 | UCUGCAGGGUUUGCUUUGAG | -0.549608986 | 0.001655472 |
| 101 | *NMT* | ENST00000543908 | *miR-1205* | 769 | 775 | UCUGCAGGGUUUGCUUUGAG | -0.549608986 | 0.001655472 |
| 102 | *NMT* | ENST00000543908 | *miR-1205* | 910 | 916 | UCUGCAGGGUUUGCUUUGAG | -0.549608986 | 0.001655472 |
| 103 | *NMT* | ENST00000543908 | *miR-1205* | 97 | 102 | UCUGCAGGGUUUGCUUUGAG | -0.549608986 | 0.001655472 |
| 104 | *NMT* | ENST00000592782 | *miR-1205* | 1009 | 1015 | UCUGCAGGGUUUGCUUUGAG | -0.549608986 | 0.001655472 |
| 105 | *NMT* | ENST00000592782 | *miR-1205* | 1550 | 1556 | UCUGCAGGGUUUGCUUUGAG | -0.549608986 | 0.001655472 |
| 106 | *NMT* | ENST00000592782 | *miR-1205* | 218 | 223 | UCUGCAGGGUUUGCUUUGAG | -0.549608986 | 0.001655472 |
| 107 | *NMT* | ENST00000592782 | *miR-1205* | 2284 | 2290 | UCUGCAGGGUUUGCUUUGAG | -0.549608986 | 0.001655472 |
| 108 | *NMT* | ENST00000592782 | *miR-1205* | 3216 | 3222 | UCUGCAGGGUUUGCUUUGAG | -0.549608986 | 0.001655472 |
| 109 | *NMT* | ENST00000592782 | *miR-1205* | 3292 | 3298 | UCUGCAGGGUUUGCUUUGAG | -0.549608986 | 0.001655472 |
| 110 | *NMT* | ENST00000592782 | *miR-1205* | 3346 | 3352 | UCUGCAGGGUUUGCUUUGAG | -0.549608986 | 0.001655472 |
| 111 | *NMT* | ENST00000592782 | *miR-1205* | 4044 | 4050 | UCUGCAGGGUUUGCUUUGAG | -0.549608986 | 0.001655472 |
| 112 | *NMT* | ENST00000592782 | *miR-1205* | 43 | 50 | UCUGCAGGGUUUGCUUUGAG | -0.549608986 | 0.001655472 |
| 113 | *NMT* | ENST00000378165 | *miR-512-3p* | 2258 | 2263 | AAGUGCUGUCAUAGCUGAGGUC | -0.545969899 | 1.80E-003 |
| 114 | *NMT* | ENST00000378165 | *miR-512-3p* | 2320 | 2326 | AAGUGCUGUCAUAGCUGAGGUC | -0.545969899 | 1.80E-003 |
| 115 | *NMT* | ENST00000585561 | *miR-512-3p* | 79 | 84 | AAGUGCUGUCAUAGCUGAGGUC | -0.545969899 | 0.001802839 |
| 116 | *NMT* | ENST00000378150 | *miR-512-3p* | 2236 | 2241 | AAGUGCUGUCAUAGCUGAGGUC | -0.545969899 | 0.001802839 |
| 117 | *NMT* | ENST00000378150 | *miR-512-3p* | 2298 | 2304 | AAGUGCUGUCAUAGCUGAGGUC | -0.545969899 | 0.001802839 |
| 118 | *NMT* | ENST00000590114 | *miR-512-3p* | 79 | 84 | AAGUGCUGUCAUAGCUGAGGUC | -0.545969899 | 0.001802839 |
| 119 | *NMT* | ENST00000588975 | *miR-512-3p* | 107 | 112 | AAGUGCUGUCAUAGCUGAGGUC | -0.545969899 | 1.80E-003 |
| 120 | *NMT* | ENST00000587014 | *miR-512-3p* | 110 | 115 | AAGUGCUGUCAUAGCUGAGGUC | -0.545969899 | 0.001802839 |
| 121 | *NMT* | ENST00000258960 | *miR-512-3p* | 101 | 106 | AAGUGCUGUCAUAGCUGAGGUC | -0.545969899 | 0.001802839 |
| 122 | *NMT* | ENST00000258960 | *miR-512-3p* | 1133 | 1138 | AAGUGCUGUCAUAGCUGAGGUC | -0.545969899 | 0.001802839 |
| 123 | *NMT* | ENST00000543908 | *miR-512-3p* | 93 | 98 | AAGUGCUGUCAUAGCUGAGGUC | -0.545969899 | 0.001802839 |
| 124 | *NMT* | ENST00000592782 | *miR-512-3p* | 1246 | 1251 | AAGUGCUGUCAUAGCUGAGGUC | -0.545969899 | 0.001802839 |
| 125 | *NMT* | ENST00000592782 | *miR-512-3p* | 214 | 219 | AAGUGCUGUCAUAGCUGAGGUC | -0.545969899 | 0.001802839 |
| 126 | *NMT* | ENST00000591931 | *miR-330-3p* | 159 | 165 | GCAAAGCACACGGCCUGCAGAGA | -0.541276179 | 2.01E-003 |
| 127 | *NMT* | ENST00000591931 | *miR-330-3p* | 233 | 239 | GCAAAGCACACGGCCUGCAGAGA | -0.541276179 | 0.002009633 |
| 128 | *NMT* | ENST00000585561 | *miR-330-3p* | 571 | 577 | GCAAAGCACACGGCCUGCAGAGA | -0.541276179 | 2.01E-003 |
| 129 | *NMT* | ENST00000588455 | *miR-330-3p* | 146 | 152 | GCAAAGCACACGGCCUGCAGAGA | -0.541276179 | 2.01E-003 |
| 130 | *NMT* | ENST00000590114 | *miR-330-3p* | 473 | 479 | GCAAAGCACACGGCCUGCAGAGA | -0.541276179 | 2.01E-003 |
| 131 | *NMT* | ENST00000587014 | *miR-330-3p* | 730 | 736 | GCAAAGCACACGGCCUGCAGAGA | -0.541276179 | 0.002009633 |
| 132 | *NMT* | ENST00000258960 | *miR-330-3p* | 2079 | 2085 | GCAAAGCACACGGCCUGCAGAGA | -0.541276179 | 0.002009633 |
| 133 | *NMT* | ENST00000258960 | *miR-330-3p* | 3404 | 3410 | GCAAAGCACACGGCCUGCAGAGA | -0.541276179 | 0.002009633 |
| 134 | *NMT* | ENST00000258960 | *miR-330-3p* | 495 | 501 | GCAAAGCACACGGCCUGCAGAGA | -0.541276179 | 0.002009633 |
| 135 | *NMT* | ENST00000592782 | *miR-330-3p* | 2192 | 2198 | GCAAAGCACACGGCCUGCAGAGA | -0.541276179 | 2.01E-003 |
| 136 | *NMT* | ENST00000592782 | *miR-330-3p* | 3517 | 3523 | GCAAAGCACACGGCCUGCAGAGA | -0.541276179 | 0.002009633 |
| 137 | *NMT* | ENST00000592782 | *miR-330-3p* | 608 | 614 | GCAAAGCACACGGCCUGCAGAGA | -0.541276179 | 0.002009633 |
| 138 | *NMT* | ENST00000590310 | *miR-127-3p* | 193 | 199 | UCGGAUCCGUCUGAGCUUGGCU | -0.536313774 | 0.002250319 |
| 139 | *NMT* | ENST00000588455 | *miR-127-3p* | 458 | 464 | UCGGAUCCGUCUGAGCUUGGCU | -0.536313774 | 2.25E-003 |
| 140 | *NMT* | ENST00000258960 | *miR-127-3p* | 807 | 813 | UCGGAUCCGUCUGAGCUUGGCU | -0.536313774 | 0.002250319 |
| 141 | *NMT* | ENST00000543908 | *miR-127-3p* | 680 | 686 | UCGGAUCCGUCUGAGCUUGGCU | -0.536313774 | 0.002250319 |
| 142 | *NMT* | ENST00000592782 | *miR-127-3p* | 920 | 926 | UCGGAUCCGUCUGAGCUUGGCU | -0.536313774 | 0.002250319 |
| 143 | *NMT* | ENST00000590310 | *miR-652-5p* | 176 | 182 | CAACCCUAGGAGAGGGUGCCAUUCA | -0.536211912 | 2.26E-003 |
| 144 | *NMT* | ENST00000588455 | *miR-652-5p* | 441 | 447 | CAACCCUAGGAGAGGGUGCCAUUCA | -0.536211912 | 2.26E-003 |
| 145 | *NMT* | ENST00000590114 | *miR-652-5p* | 651 | 657 | CAACCCUAGGAGAGGGUGCCAUUCA | -0.536211912 | 0.00225551 |
| 146 | *NMT* | ENST00000466201 | *miR-652-5p* | 414 | 419 | CAACCCUAGGAGAGGGUGCCAUUCA | -0.536211912 | 0.00225551 |
| 147 | *NMT* | ENST00000258960 | *miR-652-5p* | 790 | 796 | CAACCCUAGGAGAGGGUGCCAUUCA | -0.536211912 | 0.00225551 |
| 148 | *NMT* | ENST00000543908 | *miR-652-5p* | 663 | 669 | CAACCCUAGGAGAGGGUGCCAUUCA | -0.536211912 | 0.00225551 |
| 149 | *NMT* | ENST00000543908 | *miR-652-5p* | 947 | 953 | CAACCCUAGGAGAGGGUGCCAUUCA | -0.536211912 | 0.00225551 |
| 150 | *NMT* | ENST00000592782 | *miR-652-5p* | 903 | 909 | CAACCCUAGGAGAGGGUGCCAUUCA | -0.536211912 | 0.00225551 |
| 151 | *NMT* | ENST00000378165 | *miR-3174* | 1022 | 1027 | UAGUGAGUUAGAGAUGCAGAGCC | -0.5353466 | 0.002300025 |
| 152 | *NMT* | ENST00000378165 | *miR-3174* | 2261 | 2267 | UAGUGAGUUAGAGAUGCAGAGCC | -0.5353466 | 0.002300025 |
| 153 | *NMT* | ENST00000378150 | *miR-3174* | 1000 | 1005 | UAGUGAGUUAGAGAUGCAGAGCC | -0.5353466 | 0.002300025 |
| 154 | *NMT* | ENST00000378150 | *miR-3174* | 2239 | 2245 | UAGUGAGUUAGAGAUGCAGAGCC | -0.5353466 | 0.002300025 |
| 155 | *NMT* | ENST00000466201 | *miR-3174* | 233 | 239 | UAGUGAGUUAGAGAUGCAGAGCC | -0.5353466 | 0.002300025 |
| 156 | *NMT* | ENST00000466201 | *miR-3174* | 673 | 678 | UAGUGAGUUAGAGAUGCAGAGCC | -0.5353466 | 0.002300025 |
| 157 | *NMT* | ENST00000587120 | *miR-3174* | 537 | 542 | UAGUGAGUUAGAGAUGCAGAGCC | -0.5353466 | 0.002300025 |
| 158 | *NMT* | ENST00000587670 | *miR-3174* | 260 | 265 | UAGUGAGUUAGAGAUGCAGAGCC | -0.5353466 | 0.002300025 |
| 159 | *NMT* | ENST00000258960 | *miR-3174* | 3727 | 3732 | UAGUGAGUUAGAGAUGCAGAGCC | -0.5353466 | 0.002300025 |
| 160 | *NMT* | ENST00000258960 | *miR-3174* | 4850 | 4855 | UAGUGAGUUAGAGAUGCAGAGCC | -0.5353466 | 0.002300025 |
| 161 | *NMT* | ENST00000592782 | *miR-3174* | 3840 | 3845 | UAGUGAGUUAGAGAUGCAGAGCC | -0.5353466 | 0.002300025 |
| 162 | *NMT* | ENST00000592782 | *miR-3174* | 4963 | 4968 | UAGUGAGUUAGAGAUGCAGAGCC | -0.5353466 | 2.30E-003 |
| 163 | *NMT* | ENST00000378165 | *miR-1246* | 2013 | 2018 | AAUGGAUUUUUGGAGCAGG | -0.535334386 | 0.002300659 |
| 164 | *NMT* | ENST00000590310 | *miR-1246* | 101 | 107 | AAUGGAUUUUUGGAGCAGG | -0.535334386 | 0.002300659 |
| 165 | *NMT* | ENST00000585561 | *miR-1246* | 791 | 797 | AAUGGAUUUUUGGAGCAGG | -0.535334386 | 0.002300659 |
| 166 | *NMT* | ENST00000378150 | *miR-1246* | 1991 | 1996 | AAUGGAUUUUUGGAGCAGG | -0.535334386 | 0.002300659 |
| 167 | *NMT* | ENST00000588455 | *miR-1246* | 366 | 372 | AAUGGAUUUUUGGAGCAGG | -0.535334386 | 0.002300659 |
| 168 | *NMT* | ENST00000587120 | *miR-1246* | 479 | 484 | AAUGGAUUUUUGGAGCAGG | -0.535334386 | 0.002300659 |
| 169 | *NMT* | ENST00000258960 | *miR-1246* | 4792 | 4797 | AAUGGAUUUUUGGAGCAGG | -0.535334386 | 0.002300659 |
| 170 | *NMT* | ENST00000258960 | *miR-1246* | 715 | 721 | AAUGGAUUUUUGGAGCAGG | -0.535334386 | 2.30E-003 |
| 171 | *NMT* | ENST00000543908 | *miR-1246* | 588 | 594 | AAUGGAUUUUUGGAGCAGG | -0.535334386 | 0.002300659 |
| 172 | *NMT* | ENST00000592782 | *miR-1246* | 4905 | 4910 | AAUGGAUUUUUGGAGCAGG | -0.535334386 | 0.002300659 |
| 173 | *NMT* | ENST00000592782 | *miR-1246* | 828 | 834 | AAUGGAUUUUUGGAGCAGG | -0.535334386 | 0.002300659 |
| 174 | *NMT* | ENST00000378165 | *miR-1278* | 1566 | 1572 | UAGUACUGUGCAUAUCAUCUAU | -0.525529739 | 0.002860758 |
| 175 | *NMT* | ENST00000378150 | *miR-1278* | 1544 | 1550 | UAGUACUGUGCAUAUCAUCUAU | -0.525529739 | 0.002860758 |
| 176 | *NMT* | ENST00000378165 | *miR-599* | 1122 | 1128 | GUUGUGUCAGUUUAUCAAAC | -0.520143263 | 3.22E-003 |
| 177 | *NMT* | ENST00000378150 | *miR-599* | 1100 | 1106 | GUUGUGUCAGUUUAUCAAAC | -0.520143263 | 0.003215787 |
| 178 | *NMT* | ENST00000466201 | *miR-599* | 241 | 246 | GUUGUGUCAGUUUAUCAAAC | -0.520143263 | 0.003215787 |
| 179 | *NMT* | ENST00000258960 | *miR-599* | 3482 | 3488 | GUUGUGUCAGUUUAUCAAAC | -0.520143263 | 0.003215787 |
| 180 | *NMT* | ENST00000592782 | *miR-599* | 3595 | 3601 | GUUGUGUCAGUUUAUCAAAC | -0.520143263 | 0.003215787 |
| 181 | *NMT* | ENST00000587014 | *miR-346* | 335 | 340 | UGUCUGCCCGCAUGCCUGCCUCU | -0.515598853 | 0.003544184 |
| 182 | *NMT* | ENST00000258960 | *miR-346* | 1957 | 1964 | UGUCUGCCCGCAUGCCUGCCUCU | -0.515598853 | 0.003544184 |
| 183 | *NMT* | ENST00000258960 | *miR-346* | 2227 | 2232 | UGUCUGCCCGCAUGCCUGCCUCU | -0.515598853 | 0.003544184 |
| 184 | *NMT* | ENST00000258960 | *miR-346* | 3181 | 3186 | UGUCUGCCCGCAUGCCUGCCUCU | -0.515598853 | 0.003544184 |
| 185 | *NMT* | ENST00000592782 | *miR-346* | 2070 | 2077 | UGUCUGCCCGCAUGCCUGCCUCU | -0.515598853 | 0.003544184 |
| 186 | *NMT* | ENST00000592782 | *miR-346* | 2340 | 2345 | UGUCUGCCCGCAUGCCUGCCUCU | -0.515598853 | 0.003544184 |
| 187 | *NMT* | ENST00000592782 | *miR-346* | 3294 | 3299 | UGUCUGCCCGCAUGCCUGCCUCU | -0.515598853 | 0.003544184 |
| 188 | *NMT* | ENST00000592782 | *miR-346* | 46 | 51 | UGUCUGCCCGCAUGCCUGCCUCU | -0.515598853 | 0.003544184 |
| 189 | *NMT* | ENST00000378165 | *miR-543* | 1749 | 1756 | AAACAUUCGCGGUGCACUUCUU | -0.514125633 | 3.66E-003 |
| 190 | *NMT* | ENST00000378165 | *miR-543* | 2749 | 2755 | AAACAUUCGCGGUGCACUUCUU | -0.514125633 | 3.66E-003 |
| 191 | *NMT* | ENST00000378150 | *miR-543* | 1727 | 1734 | AAACAUUCGCGGUGCACUUCUU | -0.514125633 | 0.003656649 |
| 192 | *NMT* | ENST00000466201 | *miR-543* | 767 | 772 | AAACAUUCGCGGUGCACUUCUU | -0.514125633 | 0.003656649 |
| 193 | *NMT* | ENST00000378165 | *miR-548p* | 1934 | 1940 | UAGCAAAAACUGCAGUUACUUU | -0.511290642 | 0.003881714 |
| 194 | *NMT* | ENST00000378150 | *miR-548p* | 1912 | 1918 | UAGCAAAAACUGCAGUUACUUU | -0.511290642 | 0.003881714 |
| 195 | *NMT* | ENST00000587014 | *miR-548p* | 733 | 738 | UAGCAAAAACUGCAGUUACUUU | -0.511290642 | 0.003881714 |
| 196 | *NMT* | ENST00000258960 | *miR-548p* | 2360 | 2366 | UAGCAAAAACUGCAGUUACUUU | -0.511290642 | 0.003881714 |
| 197 | *NMT* | ENST00000258960 | *miR-548p* | 2888 | 2894 | UAGCAAAAACUGCAGUUACUUU | -0.511290642 | 0.003881714 |
| 198 | *NMT* | ENST00000258960 | *miR-548p* | 3303 | 3308 | UAGCAAAAACUGCAGUUACUUU | -0.511290642 | 0.003881714 |
| 199 | *NMT* | ENST00000592782 | *miR-548p* | 2473 | 2479 | UAGCAAAAACUGCAGUUACUUU | -0.511290642 | 0.003881714 |
| 200 | *NMT* | ENST00000592782 | *miR-548p* | 3001 | 3007 | UAGCAAAAACUGCAGUUACUUU | -0.511290642 | 0.003881714 |
| 201 | *NMT* | ENST00000592782 | *miR-548p* | 3416 | 3421 | UAGCAAAAACUGCAGUUACUUU | -0.511290642 | 0.003881714 |
| 202 | *NMT* | ENST00000466201 | *miR-671-5p* | 163 | 168 | AGGAAGCCCUGGAGGGGCUGGAG | -0.510501129 | 0.003946469 |
| 203 | *NMT* | ENST00000378165 | *miR-3197* | 57 | 62 | GGAGGCGCAGGCUCGGAAAGGCG | -0.508507602 | 0.004114112 |
| 204 | *NMT* | ENST00000258960 | *miR-4264* | 3324 | 3330 | ACUCAGUCAUGGUCAUU | -0.506628644 | 0.004277672 |
| 205 | *NMT* | ENST00000592782 | *miR-4264* | 3437 | 3443 | ACUCAGUCAUGGUCAUU | -0.506628644 | 0.004277672 |
| 206 | *NMT* | ENST00000378165 | *miR-137* | 2124 | 2130 | UUAUUGCUUAAGAAUACGCGUAG | -0.506096621 | 0.004324983 |
| 207 | *NMT* | ENST00000378165 | *miR-137* | 369 | 374 | UUAUUGCUUAAGAAUACGCGUAG | -0.506096621 | 0.004324983 |
| 208 | *NMT* | ENST00000478580 | *miR-137* | 409 | 414 | UUAUUGCUUAAGAAUACGCGUAG | -0.506096621 | 0.004324983 |
| 209 | *NMT* | ENST00000585561 | *miR-137* | 291 | 297 | UUAUUGCUUAAGAAUACGCGUAG | -0.506096621 | 0.004324983 |
| 210 | *NMT* | ENST00000378150 | *miR-137* | 2102 | 2108 | UUAUUGCUUAAGAAUACGCGUAG | -0.506096621 | 0.004324983 |
| 211 | *NMT* | ENST00000378150 | *miR-137* | 347 | 352 | UUAUUGCUUAAGAAUACGCGUAG | -0.506096621 | 0.004324983 |
| 212 | *NMT* | ENST00000466201 | *miR-137* | 1129 | 1136 | UUAUUGCUUAAGAAUACGCGUAG | -0.506096621 | 0.004324983 |
| 213 | *NMT* | ENST00000466201 | *miR-137* | 248 | 253 | UUAUUGCUUAAGAAUACGCGUAG | -0.506096621 | 0.004324983 |
| 214 | *NMT* | ENST00000592654 | *miR-137* | 160 | 166 | UUAUUGCUUAAGAAUACGCGUAG | -0.506096621 | 4.32E-003 |
| 215 | *NMT* | ENST00000587014 | *miR-137* | 67 | 73 | UUAUUGCUUAAGAAUACGCGUAG | -0.506096621 | 0.004324983 |
| 216 | *NMT* | ENST00000258960 | *miR-137* | 1090 | 1096 | UUAUUGCUUAAGAAUACGCGUAG | -0.506096621 | 0.004324983 |
| 217 | *NMT* | ENST00000258960 | *miR-137* | 2205 | 2211 | UUAUUGCUUAAGAAUACGCGUAG | -0.506096621 | 0.004324983 |
| 218 | *NMT* | ENST00000258960 | *miR-137* | 3286 | 3291 | UUAUUGCUUAAGAAUACGCGUAG | -0.506096621 | 0.004324983 |
| 219 | *NMT* | ENST00000592782 | *miR-137* | 1203 | 1209 | UUAUUGCUUAAGAAUACGCGUAG | -0.506096621 | 4.32E-003 |
| 220 | *NMT* | ENST00000592782 | *miR-137* | 2318 | 2324 | UUAUUGCUUAAGAAUACGCGUAG | -0.506096621 | 4.32E-003 |
| 221 | *NMT* | ENST00000592782 | *miR-137* | 3399 | 3404 | UUAUUGCUUAAGAAUACGCGUAG | -0.506096621 | 0.004324983 |
