# Supplementary material for: In silico identification of microRNAs predicted to regulate N-myristoyltransferase and Methionine Aminopeptidase 2 functions in cancer and infectious diseases
Source: PLoS One. 2018 Mar 26;13(3):e0194612. doi: 10.1371/journal.pone.0194612 (PMC5868815; doi:10.1371/journal.pone.0194612)
Supplement: S2 Table — (DOC) [file pone.0194612.s002.doc]

**S2 Table. Putative microRNAs (miRNAs) that target Methionine aminopeptidase 2** ***(METAP2)* gene**

|  | **Gene name** | **Ensemble Transcript** | **Targeting miRNA** | **Start position** | **End position** | **K-mer seed** | **PCC** | **P-value** |
| --- | --- | --- | --- | --- | --- | --- | --- | --- |
| 1 | *METAP2* | ENST00000535095 | *miR-330-3p* | 2032 | 2037 | GCAAAGCACACGGCCUGCAGAGA | -0.73 | 3.76E-06 |
| 2 | *METAP2* | ENST00000535095 | *miR-330-3p* | 2329 | 2336 | GCAAAGCACACGGCCUGCAGAGA | -0.73 | 3.76E-06 |
| 3 | *METAP2* | ENST00000323666 | *miR-330-3p* | 1705 | 1710 | GCAAAGCACACGGCCUGCAGAGA | -0.73 | 3.76E-06 |
| 4 | *METAP2* | ENST00000323666 | *miR-330-3p* | 2002 | 2009 | GCAAAGCACACGGCCUGCAGAGA | -0.73 | 3.76E-06 |
| 5 | *METAP2* | ENST00000323666 | *miR-421* | 2931 | 2937 | AUCAACAGACAUUAAUUGGGCGC | -0.73 | 4.33E-06 |
| 6 | *METAP2* | ENST00000546753 | *miR-330-3p* | 1506 | 1511 | GCAAAGCACACGGCCUGCAGAGA | -0.73 | 3.76E-06 |
| 7 | *METAP2* | ENST00000546753 | *miR-330-3p* | 1803 | 1810 | GCAAAGCACACGGCCUGCAGAGA | -0.73 | 3.76E-06 |
| 8 | *METAP2* | ENST00000261220 | *miR-330-3p* | 1437 | 1442 | GCAAAGCACACGGCCUGCAGAGA | -0.73 | 3.76E-06 |
| 9 | *METAP2* | ENST00000261220 | *miR-330-3p* | 1734 | 1741 | GCAAAGCACACGGCCUGCAGAGA | -0.73 | 3.76E-06 |
| 10 | *METAP2* | ENST00000550777 | *miR-330-3p* | 1393 | 1398 | GCAAAGCACACGGCCUGCAGAGA | -0.73 | 3.76E-06 |
| 11 | *METAP2* | ENST00000550777 | *miR-330-3p* | 1690 | 1697 | GCAAAGCACACGGCCUGCAGAGA | -0.73 | 3.76E-06 |
| 12 | *METAP2* | ENST00000551840 | *miR-330-3p* | 1491 | 1496 | GCAAAGCACACGGCCUGCAGAGA | -0.73 | 3.76E-06 |
| 13 | *METAP2* | ENST00000551840 | *miR-330-3p* | 1788 | 1795 | GCAAAGCACACGGCCUGCAGAGA | -0.73 | 3.76E-06 |
| 14 | *METAP2* | ENST00000323666 | *miR-409-3p* | 2393 | 2398 | GAAUGUUGCUCGGUGAACCCCU | -0.72 | 7.29E-06 |
| 15 | *METAP2* | ENST00000323666 | *miR-409-3p* | 2656 | 2662 | GAAUGUUGCUCGGUGAACCCCU | -0.72 | 7.29E-06 |
| 16 | *METAP2* | ENST00000535095 | *miR-139-3p* | 328 | 335 | UGGAGACGCGGCCCUGUUGGAGU | -0.69 | 2.06E-05 |
| 17 | *METAP2* | ENST00000323666 | *miR-139-3p* | 3121 | 3127 | UGGAGACGCGGCCCUGUUGGAGU | -0.69 | 2.06E-05 |
| 18 | *METAP2* | ENST00000535095 | *miR-1246* | 1881 | 1887 | AAUGGAUUUUUGGAGCAGG | -0.67 | 4.36E-05 |
| 19 | *METAP2* | ENST00000535095 | *miR-1246* | 2110 | 2115 | AAUGGAUUUUUGGAGCAGG | -0.67 | 4.36E-05 |
| 20 | *METAP2* | ENST00000535095 | *miR-1246* | 2394 | 2400 | AAUGGAUUUUUGGAGCAGG | -0.67 | 4.36E-05 |
| 21 | *METAP2* | ENST00000323666 | *miR-1246* | 1554 | 1560 | AAUGGAUUUUUGGAGCAGG | -0.67 | 4.36E-05 |
| 22 | *METAP2* | ENST00000323666 | *miR-1246* | 1783 | 1788 | AAUGGAUUUUUGGAGCAGG | -0.67 | 4.36E-05 |
| 23 | *METAP2* | ENST00000323666 | *miR-1246* | 2067 | 2073 | AAUGGAUUUUUGGAGCAGG | -0.67 | 4.36E-05 |
| 24 | *METAP2* | ENST00000546753 | *miR-1246* | 1355 | 1361 | AAUGGAUUUUUGGAGCAGG | -0.67 | 4.36E-05 |
| 25 | *METAP2* | ENST00000546753 | *miR-1246* | 1584 | 1589 | AAUGGAUUUUUGGAGCAGG | -0.67 | 4.36E-05 |
| 26 | *METAP2* | ENST00000261220 | *miR-1246* | 1286 | 1292 | AAUGGAUUUUUGGAGCAGG | -0.67 | 4.36E-05 |
| 27 | *METAP2* | ENST00000261220 | *miR-1246* | 1515 | 1520 | AAUGGAUUUUUGGAGCAGG | -0.67 | 4.36E-05 |
| 28 | *METAP2* | ENST00000261220 | *miR-1246* | 1799 | 1805 | AAUGGAUUUUUGGAGCAGG | -0.67 | 4.36E-05 |
| 29 | *METAP2* | ENST00000550777 | *miR-1246* | 1242 | 1248 | AAUGGAUUUUUGGAGCAGG | -0.67 | 4.36E-05 |
| 30 | *METAP2* | ENST00000550777 | *miR-1246* | 1471 | 1476 | AAUGGAUUUUUGGAGCAGG | -0.67 | 4.36E-05 |
| 31 | *METAP2* | ENST00000550777 | *miR-1246* | 1755 | 1761 | AAUGGAUUUUUGGAGCAGG | -0.67 | 4.36E-05 |
| 32 | *METAP2* | ENST00000551840 | *miR-1246* | 1340 | 1346 | AAUGGAUUUUUGGAGCAGG | -0.67 | 4.36E-05 |
| 33 | *METAP2* | ENST00000551840 | *miR-1246* | 1569 | 1574 | AAUGGAUUUUUGGAGCAGG | -0.67 | 4.36E-05 |
| 34 | *METAP2* | ENST00000551840 | *miR-1246* | 1853 | 1859 | AAUGGAUUUUUGGAGCAGG | -0.67 | 4.36E-05 |
| 35 | *METAP2* | ENST00000535095 | *miR-671-5p* | 1740 | 1747 | AGGAAGCCCUGGAGGGGCUGGAG | -0.65 | 8.66E-05 |
| 36 | *METAP2* | ENST00000535095 | *miR-671-5p* | 575 | 580 | AGGAAGCCCUGGAGGGGCUGGAG | -0.65 | 8.66E-05 |
| 37 | *METAP2* | ENST00000535095 | *miR-671-5p* | 91 | 97 | AGGAAGCCCUGGAGGGGCUGGAG | -0.65 | 8.66E-05 |
| 38 | *METAP2* | ENST00000323666 | *miR-671-5p* | 1413 | 1420 | AGGAAGCCCUGGAGGGGCUGGAG | -0.65 | 8.66E-05 |
| 39 | *METAP2* | ENST00000323666 | *miR-671-5p* | 144 | 149 | AGGAAGCCCUGGAGGGGCUGGAG | -0.65 | 8.66E-05 |
| 40 | *METAP2* | ENST00000546753 | *miR-671-5p* | 1214 | 1221 | AGGAAGCCCUGGAGGGGCUGGAG | -0.65 | 8.66E-05 |
| 41 | *METAP2* | ENST00000261220 | *miR-671-5p* | 1145 | 1152 | AGGAAGCCCUGGAGGGGCUGGAG | -0.65 | 8.66E-05 |
| 42 | *METAP2* | ENST00000550777 | *miR-671-5p* | 1101 | 1108 | AGGAAGCCCUGGAGGGGCUGGAG | -0.65 | 8.66E-05 |
| 43 | *METAP2* | ENST00000551840 | *miR-671-5p* | 1199 | 1206 | AGGAAGCCCUGGAGGGGCUGGAG | -0.65 | 8.66E-05 |
| 44 | *METAP2* | ENST00000535095 | *miR-362-5p* | 1908 | 1913 | AAUCCUUGGAACCUAGGUGUGAGU | -0.64 | 1.57E-04 |
| 45 | *METAP2* | ENST00000535095 | *miR-362-5p* | 607 | 612 | AAUCCUUGGAACCUAGGUGUGAGU | -0.64 | 1.57E-04 |
| 46 | *METAP2* | ENST00000323666 | *miR-362-5p* | 1581 | 1586 | AAUCCUUGGAACCUAGGUGUGAGU | -0.64 | 1.57E-04 |
| 47 | *METAP2* | ENST00000323666 | *miR-362-5p* | 176 | 181 | AAUCCUUGGAACCUAGGUGUGAGU | -0.64 | 1.57E-04 |
| 48 | *METAP2* | ENST00000546753 | *miR-362-5p* | 1382 | 1387 | AAUCCUUGGAACCUAGGUGUGAGU | -0.64 | 1.57E-04 |
| 49 | *METAP2* | ENST00000546753 | *miR-362-5p* | 46 | 51 | AAUCCUUGGAACCUAGGUGUGAGU | -0.64 | 1.57E-04 |
| 50 | *METAP2* | ENST00000261220 | *miR-362-5p* | 1313 | 1318 | AAUCCUUGGAACCUAGGUGUGAGU | -0.64 | 1.57E-04 |
| 51 | *METAP2* | ENST00000549502 | *miR-362-5p* | 285 | 291 | AAUCCUUGGAACCUAGGUGUGAGU | -0.64 | 1.57E-04 |
| 52 | *METAP2* | ENST00000550777 | *miR-362-5p* | 1269 | 1274 | AAUCCUUGGAACCUAGGUGUGAGU | -0.64 | 1.57E-04 |
| 53 | *METAP2* | ENST00000551840 | *miR-362-5p* | 1367 | 1372 | AAUCCUUGGAACCUAGGUGUGAGU | -0.64 | 1.57E-04 |
| 54 | *METAP2* | ENST00000535095 | *miR-4306* | 102 | 109 | UGGAGAGAAAGGCAGUA | -0.63 | 1.77E-04 |
| 55 | *METAP2* | ENST00000535095 | *miR-532-3p* | 1824 | 1830 | CCUCCCACACCCAAGGCUUGCA | -0.61 | 3.37E-04 |
| 56 | *METAP2* | ENST00000323666 | *miR-1244* | 2769 | 2774 | AAGUAGUUGGUUUGUAUGAGAUGGUU | -0.61 | 3.18E-04 |
| 57 | *METAP2* | ENST00000323666 | *miR-532-3p* | 1497 | 1503 | CCUCCCACACCCAAGGCUUGCA | -0.61 | 3.37E-04 |
| 58 | *METAP2* | ENST00000323666 | *miR-532-3p* | 3389 | 3394 | CCUCCCACACCCAAGGCUUGCA | -0.61 | 3.37E-04 |
| 59 | *METAP2* | ENST00000546753 | *miR-532-3p* | 1298 | 1304 | CCUCCCACACCCAAGGCUUGCA | -0.61 | 3.37E-04 |
| 60 | *METAP2* | ENST00000261220 | *miR-532-3p* | 1229 | 1235 | CCUCCCACACCCAAGGCUUGCA | -0.61 | 3.37E-04 |
| 61 | *METAP2* | ENST00000550777 | *miR-532-3p* | 1185 | 1191 | CCUCCCACACCCAAGGCUUGCA | -0.61 | 3.37E-04 |
| 62 | *METAP2* | ENST00000551840 | *miR-532-3p* | 1283 | 1289 | CCUCCCACACCCAAGGCUUGCA | -0.61 | 3.37E-04 |
| 63 | *METAP2* | ENST00000535095 | *miR-107* | 1344 | 1349 | AGCAGCAUUGUACAGGGCUAUCA | -0.6 | 4.60E-04 |
| 64 | *METAP2* | ENST00000535095 | *miR-665* | 2388 | 2394 | ACCAGGAGGCUGAGGCCCCU | -0.6 | 4.54E-04 |
| 65 | *METAP2* | ENST00000323666 | *miR-107* | 913 | 918 | AGCAGCAUUGUACAGGGCUAUCA | -0.6 | 4.60E-04 |
| 66 | *METAP2* | ENST00000323666 | *miR-665* | 2061 | 2067 | ACCAGGAGGCUGAGGCCCCU | -0.6 | 4.54E-04 |
| 67 | *METAP2* | ENST00000546753 | *miR-107* | 714 | 719 | AGCAGCAUUGUACAGGGCUAUCA | -0.6 | 4.60E-04 |
| 68 | *METAP2* | ENST00000261220 | *miR-107* | 645 | 650 | AGCAGCAUUGUACAGGGCUAUCA | -0.6 | 4.60E-04 |
| 69 | *METAP2* | ENST00000261220 | *miR-665* | 1793 | 1799 | ACCAGGAGGCUGAGGCCCCU | -0.6 | 4.54E-04 |
| 70 | *METAP2* | ENST00000550777 | *miR-107* | 601 | 606 | AGCAGCAUUGUACAGGGCUAUCA | -0.6 | 4.60E-04 |
| 71 | *METAP2* | ENST00000550777 | *miR-665* | 1749 | 1755 | ACCAGGAGGCUGAGGCCCCU | -0.6 | 4.54E-04 |
| 72 | *METAP2* | ENST00000551840 | *miR-107* | 699 | 704 | AGCAGCAUUGUACAGGGCUAUCA | -0.6 | 4.60E-04 |
| 73 | *METAP2* | ENST00000551840 | *miR-665* | 1847 | 1853 | ACCAGGAGGCUGAGGCCCCU | -0.6 | 4.54E-04 |
| 74 | *METAP2* | ENST00000549808 | *miR-107* | 439 | 444 | AGCAGCAUUGUACAGGGCUAUCA | -0.6 | 4.60E-04 |
| 75 | *METAP2* | ENST00000535095 | *miR-654-3p* | 1613 | 1619 | UAUGUCUGCUGACCAUCACCUU | -0.58 | 8.40E-04 |
| 76 | *METAP2* | ENST00000535095 | *miR-654-3p* | 2415 | 2420 | UAUGUCUGCUGACCAUCACCUU | -0.58 | 8.40E-04 |
| 77 | *METAP2* | ENST00000323666 | *miR-324-5p* | 2908 | 2914 | CGCAUCCCCUAGGGCAUUGGUGU | -0.58 | 8.70E-04 |
| 78 | *METAP2* | ENST00000323666 | *miR-654-3p* | 1182 | 1188 | UAUGUCUGCUGACCAUCACCUU | -0.58 | 8.40E-04 |
| 79 | *METAP2* | ENST00000323666 | *miR-654-3p* | 2088 | 2093 | UAUGUCUGCUGACCAUCACCUU | -0.58 | 8.40E-04 |
| 80 | *METAP2* | ENST00000323666 | *miR-654-3p* | 2179 | 2184 | UAUGUCUGCUGACCAUCACCUU | -0.58 | 8.40E-04 |
| 81 | *METAP2* | ENST00000546753 | *miR-654-3p* | 983 | 989 | UAUGUCUGCUGACCAUCACCUU | -0.58 | 8.40E-04 |
| 82 | *METAP2* | ENST00000261220 | *miR-654-3p* | 1820 | 1825 | UAUGUCUGCUGACCAUCACCUU | -0.58 | 8.40E-04 |
| 83 | *METAP2* | ENST00000261220 | *miR-654-3p* | 914 | 920 | UAUGUCUGCUGACCAUCACCUU | -0.58 | 8.40E-04 |
| 84 | *METAP2* | ENST00000550777 | *miR-654-3p* | 870 | 876 | UAUGUCUGCUGACCAUCACCUU | -0.58 | 8.40E-04 |
| 85 | *METAP2* | ENST00000551840 | *miR-654-3p* | 968 | 974 | UAUGUCUGCUGACCAUCACCUU | -0.58 | 8.40E-04 |
| 86 | *METAP2* | ENST00000535095 | *miR-650* | 685 | 690 | AGGAGGCAGCGCUCUCAGGAC | -0.57 | 9.32E-04 |
| 87 | *METAP2* | ENST00000323666 | *miR-650* | 254 | 259 | AGGAGGCAGCGCUCUCAGGAC | -0.57 | 9.32E-04 |
| 88 | *METAP2* | ENST00000546753 | *miR-650* | 124 | 129 | AGGAGGCAGCGCUCUCAGGAC | -0.57 | 9.32E-04 |
| 89 | *METAP2* | ENST00000261220 | *miR-650* | 55 | 60 | AGGAGGCAGCGCUCUCAGGAC | -0.57 | 9.32E-04 |
| 90 | *METAP2* | ENST00000549502 | *miR-650* | 53 | 58 | AGGAGGCAGCGCUCUCAGGAC | -0.57 | 9.32E-04 |
| 91 | *METAP2* | ENST00000553151 | *miR-650* | 53 | 58 | AGGAGGCAGCGCUCUCAGGAC | -0.57 | 9.32E-04 |
| 92 | *METAP2* | ENST00000550777 | *miR-650* | 50 | 55 | AGGAGGCAGCGCUCUCAGGAC | -0.57 | 9.32E-04 |
| 93 | *METAP2* | ENST00000551840 | *miR-650* | 43 | 48 | AGGAGGCAGCGCUCUCAGGAC | -0.57 | 9.32E-04 |
| 94 | *METAP2* | ENST00000535095 | *miR-199b-3p* | 1319 | 1324 | ACAGUAGUCUGCACAUUGGUUA | -0.55 | 1.72E-03 |
| 95 | *METAP2* | ENST00000535095 | *miR-299-3p* | 2067 | 2072 | UAUGUGGGAUGGUAAACCGCUU | -0.55 | 1.66E-03 |
| 96 | *METAP2* | ENST00000323666 | *miR-199b-3p* | 2769 | 2776 | ACAGUAGUCUGCACAUUGGUUA | -0.55 | 1.72E-03 |
| 97 | *METAP2* | ENST00000323666 | *miR-199b-3p* | 888 | 893 | ACAGUAGUCUGCACAUUGGUUA | -0.55 | 0 |
| 98 | *METAP2* | ENST00000323666 | *miR-299-3p* | 1740 | 1745 | UAUGUGGGAUGGUAAACCGCUU | -0.55 | 0 |
| 99 | *METAP2* | ENST00000323666 | *miR-299-3p* | 3106 | 3111 | UAUGUGGGAUGGUAAACCGCUU | -0.55 | 1.66E-03 |
| 100 | *METAP2* | ENST00000323666 | *miR-4319* | 2468 | 2474 | UCCCUGAGCAAAGCCAC | -0.55 | 1.55E-03 |
| 101 | *METAP2* | ENST00000323666 | *miR-4319* | 3185 | 3190 | UCCCUGAGCAAAGCCAC | -0.55 | 1.55E-03 |
| 102 | *METAP2* | ENST00000323666 | *miR-520d-5p* | 2314 | 2320 | CUACAAAGGGAAGCCCUUUC | -0.55 | 0 |
| 103 | *METAP2* | ENST00000323666 | *miR-520d-5p* | 2869 | 2874 | CUACAAAGGGAAGCCCUUUC | -0.55 | 1.63E-03 |
| 104 | *METAP2* | ENST00000323666 | *miR-520d-5p* | 2922 | 2927 | CUACAAAGGGAAGCCCUUUC | -0.55 | 1.63E-03 |
| 105 | *METAP2* | ENST00000546753 | *miR-199b-3p* | 689 | 694 | ACAGUAGUCUGCACAUUGGUUA | -0.55 | 1.72E-03 |
| 106 | *METAP2* | ENST00000546753 | *miR-299-3p* | 1541 | 1546 | UAUGUGGGAUGGUAAACCGCUU | -0.55 | 1.66E-03 |
| 107 | *METAP2* | ENST00000261220 | *miR-199b-3p* | 620 | 625 | ACAGUAGUCUGCACAUUGGUUA | -0.55 | 1.72E-03 |
| 108 | *METAP2* | ENST00000261220 | *miR-299-3p* | 1472 | 1477 | UAUGUGGGAUGGUAAACCGCUU | -0.55 | 1.66E-03 |
| 109 | *METAP2* | ENST00000550777 | *miR-199b-3p* | 576 | 581 | ACAGUAGUCUGCACAUUGGUUA | -0.55 | 0 |
| 110 | *METAP2* | ENST00000550777 | *miR-299-3p* | 1428 | 1433 | UAUGUGGGAUGGUAAACCGCUU | -0.55 | 1.66E-03 |
| 111 | *METAP2* | ENST00000551840 | *miR-199b-3p* | 674 | 679 | ACAGUAGUCUGCACAUUGGUUA | -0.55 | 0 |
| 112 | *METAP2* | ENST00000551840 | *miR-299-3p* | 1526 | 1531 | UAUGUGGGAUGGUAAACCGCUU | -0.55 | 0 |
| 113 | *METAP2* | ENST00000549808 | *miR-199b-3p* | 414 | 419 | ACAGUAGUCUGCACAUUGGUUA | -0.55 | 1.72E-03 |
| 114 | *METAP2* | ENST00000546478 | *miR-520d-5p* | 57 | 62 | CUACAAAGGGAAGCCCUUUC | -0.55 | 0 |
| 115 | *METAP2* | ENST00000323666 | *miR-140-3p* | 2123 | 2129 | UACCACAGGGUAGAACCACGG | -0.54 | 0 |
| 116 | *METAP2* | ENST00000549136 | *miR-140-3p* | 230 | 235 | UACCACAGGGUAGAACCACGG | -0.54 | 0 |
| 117 | *METAP2* | ENST00000549808 | *miR-140-3p* | 53 | 58 | UACCACAGGGUAGAACCACGG | -0.54 | 2.19E-03 |
| 118 | *METAP2* | ENST00000535095 | *miR-199a-3p* | 1319 | 1324 | ACAGUAGUCUGCACAUUGGUUA | -0.53 | 0 |
| 119 | *METAP2* | ENST00000535095 | *miR-543* | 1691 | 1697 | AAACAUUCGCGGUGCACUUCUU | -0.53 | 2.59E-03 |
| 120 | *METAP2* | ENST00000323666 | *miR-199a-3p* | 2769 | 2776 | ACAGUAGUCUGCACAUUGGUUA | -0.53 | 2.45E-03 |
| 121 | *METAP2* | ENST00000323666 | *miR-199a-3p* | 888 | 893 | ACAGUAGUCUGCACAUUGGUUA | -0.53 | 0 |
| 122 | *METAP2* | ENST00000323666 | *miR-543* | 1364 | 1370 | AAACAUUCGCGGUGCACUUCUU | -0.53 | 2.59E-03 |
| 123 | *METAP2* | ENST00000323666 | *miR-543* | 2434 | 2439 | AAACAUUCGCGGUGCACUUCUU | -0.53 | 0 |
| 124 | *METAP2* | ENST00000323666 | *miR-543* | 2819 | 2825 | AAACAUUCGCGGUGCACUUCUU | -0.53 | 0 |
| 125 | *METAP2* | ENST00000323666 | *miR-543* | 3030 | 3037 | AAACAUUCGCGGUGCACUUCUU | -0.53 | 0 |
| 126 | *METAP2* | ENST00000323666 | *miR-543* | 3094 | 3099 | AAACAUUCGCGGUGCACUUCUU | -0.53 | 0 |
| 127 | *METAP2* | ENST00000323666 | *miR-628-3p* | 2660 | 2666 | UCUAGUAAGAGUGGCAGUCGA | -0.53 | 0 |
| 128 | *METAP2* | ENST00000546753 | *miR-199a-3p* | 689 | 694 | ACAGUAGUCUGCACAUUGGUUA | -0.53 | 2.45E-03 |
| 129 | *METAP2* | ENST00000546753 | *miR-543* | 1165 | 1171 | AAACAUUCGCGGUGCACUUCUU | -0.53 | 0 |
| 130 | *METAP2* | ENST00000261220 | *miR-199a-3p* | 620 | 625 | ACAGUAGUCUGCACAUUGGUUA | -0.53 | 0 |
| 131 | *METAP2* | ENST00000261220 | *miR-543* | 1096 | 1102 | AAACAUUCGCGGUGCACUUCUU | -0.53 | 0 |
| 132 | *METAP2* | ENST00000550777 | *miR-199a-3p* | 576 | 581 | ACAGUAGUCUGCACAUUGGUUA | -0.53 | 2.45E-03 |
| 133 | *METAP2* | ENST00000550777 | *miR-543* | 1052 | 1058 | AAACAUUCGCGGUGCACUUCUU | -0.53 | 0 |
| 134 | *METAP2* | ENST00000551840 | *miR-199a-3p* | 674 | 679 | ACAGUAGUCUGCACAUUGGUUA | -0.53 | 0 |
| 135 | *METAP2* | ENST00000551840 | *miR-543* | 1150 | 1156 | AAACAUUCGCGGUGCACUUCUU | -0.53 | 2.59E-03 |
| 136 | *METAP2* | ENST00000549136 | *miR-543* | 191 | 197 | AAACAUUCGCGGUGCACUUCUU | -0.53 | 2.59E-03 |
| 137 | *METAP2* | ENST00000549808 | *miR-199a-3p* | 414 | 419 | ACAGUAGUCUGCACAUUGGUUA | -0.53 | 0 |
| 138 | *METAP2* | ENST00000549808 | *miR-543* | 20 | 25 | AAACAUUCGCGGUGCACUUCUU | -0.53 | 0 |
| 139 | *METAP2* | ENST00000535095 | *miR-1972* | 1306 | 1311 | UCAGGCCAGGCACAGUGGCUCA | -0.52 | 3.11E-03 |
| 140 | *METAP2* | ENST00000535095 | *miR-501-5p* | 1907 | 1913 | AAUCCUUUGUCCCUGGGUGAGA | -0.52 | 0 |
| 141 | *METAP2* | ENST00000535095 | *miR-501-5p* | 607 | 612 | AAUCCUUUGUCCCUGGGUGAGA | -0.52 | 3.05E-03 |
| 142 | *METAP2* | ENST00000323666 | *miR-1972* | 875 | 880 | UCAGGCCAGGCACAGUGGCUCA | -0.52 | 3.11E-03 |
| 143 | *METAP2* | ENST00000323666 | *miR-299-5p* | 3270 | 3275 | UGGUUUACCGUCCCACAUACAU | -0.52 | 3.04E-03 |
| 144 | *METAP2* | ENST00000323666 | *miR-501-5p* | 1580 | 1586 | AAUCCUUUGUCCCUGGGUGAGA | -0.52 | 3.05E-03 |
| 145 | *METAP2* | ENST00000323666 | *miR-501-5p* | 176 | 181 | AAUCCUUUGUCCCUGGGUGAGA | -0.52 | 3.05E-03 |
| 146 | *METAP2* | ENST00000546753 | *miR-1972* | 676 | 681 | UCAGGCCAGGCACAGUGGCUCA | -0.52 | 3.11E-03 |
| 147 | *METAP2* | ENST00000546753 | *miR-501-5p* | 1381 | 1387 | AAUCCUUUGUCCCUGGGUGAGA | -0.52 | 3.05E-03 |
| 148 | *METAP2* | ENST00000546753 | *miR-501-5p* | 46 | 51 | AAUCCUUUGUCCCUGGGUGAGA | -0.52 | 3.05E-03 |
| 149 | *METAP2* | ENST00000261220 | *miR-1972* | 607 | 612 | UCAGGCCAGGCACAGUGGCUCA | -0.52 | 0 |
| 150 | *METAP2* | ENST00000261220 | *miR-501-5p* | 1312 | 1318 | AAUCCUUUGUCCCUGGGUGAGA | -0.52 | 0 |
| 151 | *METAP2* | ENST00000549502 | *miR-501-5p* | 285 | 291 | AAUCCUUUGUCCCUGGGUGAGA | -0.52 | 0 |
| 152 | *METAP2* | ENST00000550777 | *miR-1972* | 563 | 568 | UCAGGCCAGGCACAGUGGCUCA | -0.52 | 0 |
| 153 | *METAP2* | ENST00000550777 | *miR-501-5p* | 1268 | 1274 | AAUCCUUUGUCCCUGGGUGAGA | -0.52 | 0 |
| 154 | *METAP2* | ENST00000551840 | *miR-1972* | 661 | 666 | UCAGGCCAGGCACAGUGGCUCA | -0.52 | 3.11E-03 |
| 155 | *METAP2* | ENST00000551840 | *miR-501-5p* | 1366 | 1372 | AAUCCUUUGUCCCUGGGUGAGA | -0.52 | 3.05E-03 |
| 156 | *METAP2* | ENST00000549808 | *miR-1972* | 401 | 406 | UCAGGCCAGGCACAGUGGCUCA | -0.52 | 3.11E-03 |
| 157 | *METAP2* | ENST00000535095 | *miR-485-5p* | 120 | 125 | AGAGGCUGGCCGUGAUGAAUUC | -0.51 | 0 |
| 158 | *METAP2* | ENST00000535095 | *miR-485-5p* | 843 | 849 | AGAGGCUGGCCGUGAUGAAUUC | -0.51 | 0 |
| 159 | *METAP2* | ENST00000323666 | *miR-485-5p* | 412 | 418 | AGAGGCUGGCCGUGAUGAAUUC | -0.51 | 0 |
| 160 | *METAP2* | ENST00000546753 | *miR-485-5p* | 282 | 288 | AGAGGCUGGCCGUGAUGAAUUC | -0.51 | 3.61E-03 |
| 161 | *METAP2* | ENST00000261220 | *miR-485-5p* | 210 | 216 | AGAGGCUGGCCGUGAUGAAUUC | -0.51 | 0 |
| 162 | *METAP2* | ENST00000549502 | *miR-485-5p* | 211 | 217 | AGAGGCUGGCCGUGAUGAAUUC | -0.51 | 0 |
| 163 | *METAP2* | ENST00000551840 | *miR-485-5p* | 198 | 204 | AGAGGCUGGCCGUGAUGAAUUC | -0.51 | 3.61E-03 |
| 164 | *METAP2* | ENST00000549136 | *miR-485-5p* | 32 | 38 | AGAGGCUGGCCGUGAUGAAUUC | -0.51 | 3.61E-03 |
